# Efficient IL-2R signaling differentially affects the stability, function, and composition of the regulatory T-cell pool

**DOI:** 10.1038/s41423-020-00599-z

**Published:** 2021-01-06

**Authors:** Marc Permanyer, Berislav Bošnjak, Silke Glage, Michaela Friedrichsen, Stefan Floess, Jochen Huehn, Gwendolyn E. Patzer, Ivan Odak, Nadine Eckert, Razieh Zargari, Laura Ospina-Quintero, Hristo Georgiev, Reinhold Förster

**Affiliations:** 1 Institute of Immunology, Hannover Medical School, Hannover, Germany; 2 Institute for Laboratory Animal Science, Hannover Medical School, Hannover, Germany; 3 Department Experimental Immunology, Helmholtz Centre for Infection Research, Braunschweig, Germany; 4 Cluster of Excellence RESIST (EXC 2155), Hannover Medical School, 30625 Hannover, Germany

**Keywords:** Regulatory T cells, IL-2R signaling, scRNA sequencing, Treg heterogeneity, Autoimmunity, Regulatory T cells

## Abstract

Signaling via interleukin-2 receptor (IL-2R) is a requisite for regulatory T (Treg) cell identity and function. However, it is not completely understood to what degree IL-2R signaling is required for Treg cell homeostasis, lineage stability and function in both resting and inflammatory conditions. Here, we characterized a spontaneous mutant mouse strain endowed with a hypomorphic Tyr129His variant of CD25, the α-chain of IL-2R, which resulted in diminished receptor expression and reduced IL-2R signaling. Under noninflammatory conditions, *Cd25*^Y129H^ mice harbored substantially lower numbers of peripheral Treg cells with stable Foxp3 expression that prevented the development of spontaneous autoimmune disease. In contrast, *Cd25*^Y129H^ Treg cells failed to efficiently induce immune suppression and lost lineage commitment in a T-cell transfer colitis model, indicating that unimpaired IL-2R signaling is critical for Treg cell function in inflammatory environments. Moreover, single-cell RNA sequencing of Treg cells revealed that impaired IL-2R signaling profoundly affected the balance of central and effector Treg cell subsets. Thus, partial loss of IL-2R signaling differentially interferes with the maintenance, heterogeneity, and suppressive function of the Treg cell pool.

## Introduction

Regulatory T cells (Treg cells), which are potent immunosuppressors, are characterized by the expression of the lineage-specification factor Foxp3.^1,2^ Efficient interleukin-2 receptor (IL-2R) signaling is essential for the suppressive activity of Treg cells and their homeostasis. These findings have been inferred from germline knockout animal model,^3,4^ neutralizing antibody,^5,6^ adoptive cell transfer,^7,8^ and in vitro studies.^9,10^ However, efforts to evaluate the requirements for IL-2R signaling in Treg cell development, peripheral homeostasis, and suppressive function have been hampered by the emergence of massive lymphocyte proliferation and lethal autoimmunity in mice carrying germline or cell type-specific mutations affecting IL-2R signaling components.^4,11–13^ Furthermore, some of the initial studies addressing Treg cell biology primarily relied on CD25 as a Treg cell marker, which made it difficult to isolate or detect these cells in IL-2R-deficient mice.

IL-2R consists of three components known as IL-2Rα (CD25), IL-2Rβ (CD122), and the common γc chain (CD132). IL-2 binds weakly to either monomers of IL-2Rα or heterodimers of IL-2Rβ/γc, but its affinity increases ~1000-fold when all three subunits form a heterotrimer. Only the heterotrimeric high-affinity receptor seems to be physiologically relevant, since *Il-2*- and *Il-2Rα*-deficient mice show similar phenotypes.^12,14^ IL-2Rβ and γc also serve as subunits of IL-15R that contributes to Treg cell differentiation.^15^ Although it is well established that IL-2 maintains peripheral Treg cells, it is not fully understood at which stage and to what degree IL-2R signaling contributes to the induction of Foxp3 expression and Treg cell lineage stability or whether this requirement differs in resting versus inflammatory conditions. For example, antibody-mediated IL-2 neutralization was found to reduce Foxp3 expression in Treg cells.^6,16^ However, IL-2-specific neutralizing antibodies act only for a short period of time, cause an immediate reduction in Treg cell numbers^6,17^ and have opposite effects on other T-cell populations,^18^ thus preventing the assessment of Treg cell stability in the context of defective IL-2R signaling. Alternatively, several reports have also shown diminished Foxp3 expression in IL-2-, IL-2Rα-, and IL-2Rβ-deficient mice.^3,4^ Since IL-2R signaling is also essential during thymic Treg cell development,^4,19^ it is difficult to ascribe the early disease symptoms found in germline knockout mice to either defective thymic Treg cell development or to the malfunctioning of extrathymic Treg cells.

In the present study, we identified mice harboring a novel spontaneous mutation within the Cd25 locus (*Cd25*^Y129H^) that markedly abrogated the surface and intracellular expression of CD25 in T cells. The reduced levels of CD25 significantly affected responsiveness to IL-2, the frequency of Treg cells and their in vitro suppressive capacity. Surprisingly, despite the defects in Treg cell maintenance, low IL-2R signaling was sufficient to preserve the lineage stability and suppressor function of Treg cells under homeostatic conditions. The unexpected lack of spontaneous autoimmune disease in *Cd25*^Y129H^ mice offered the possibility to further address the role of CD25 for stability and function of the Treg cell pool in the context of lifelong decreased IL-2R signaling. We provide evidence that—in contrast to the findings in steady-state situations—efficient IL-2R signaling is indispensable for Treg cell lineage stability and suppressor function under inflammatory conditions. Moreover, single-cell (sc) RNA sequencing revealed that continuously suboptimal IL-2R signaling profoundly affected the balance of Treg cell subsets, which were strongly shifted toward an effector phenotype in *Cd25*^Y129H^ mice.

## Materials and methods

### Animals

The following mouse strains were used: C57BL/6 (B6), B6(Cg)-Rag2tm1.1Cgn/J (Rag2^−/−^), B6.129S4-Ccr2tm1Ifc/J,^20^ and B6.129P2-Ccr2tm1Mae,^21^ which were kindly provided by Natalio Garbi (Bonn, Germany), and B6.Cg-Foxp3tm1Mal/J (Foxp3^GFP^),^22^ which was kindly provided by Bernard Malissen (Marseille, France). *Ccr2*^tm1Ifc^ mice were obtained from The Jackson Laboratory in 2008 and maintained by homozygous mating at Hannover Medical School’s Central Animal Facility without detecting physical or behavioral abnormalities. *Ccr2*^tm1Mae^ mice were purchased from Taconic in 2008 and bred under specific pathogen-free conditions in the animal facilities of the University of Bonn, Germany. *Cd25*^Y129H^ mice were derived from *Ccr2*^tm1Ifc^ mice intercrossed for at least three further generations with C57BL/6 mice and carried the WT alleles of *Ccr2* and the *Cd25*-Y129H point mutation. *Cd25*^Y129H^ mice were intercrossed with Foxp3^GFP^ mice to generate *Cd25*^Y129H^Foxp3^GFP^ mice. The homozygous *Cd25*^Y129H^ mice and cohoused littermate WT controls (*Cd25*^wt^) used in all experiments were obtained by intercrossing heterozygous *Cd25*^Y129H^ (*Cd25*^wt/Y129H^) mice. For mouse genotyping, genomic DNA was extracted from tail biopsies by lysis with 200 mM NaCl, 100 mM Tris-HCl pH 8.5, 5 mM EDTA, 0.2% SDS, and 100 μg/ml Proteinase K followed by isopropanol precipitation. PCR was performed using DreamTaq Green PCR Master Mix (Thermo Scientific), and PCR products were purified using a QIAquick PCR Purification Kit (Qiagen) following the manufacturer’s recommendations. Sanger DNA sequencing was performed by GATC (Konstanz, Germany). Mice were bred under specific pathogen-free conditions at the Central Animal Facility at Hannover Medical School. Both males and females were used equally throughout the experiments. All experiments were conducted in accordance with the local animal welfare regulations reviewed by the institutional review board and the Niedersächsisches Landesamt für Verbraucherschutz und Lebensmittelsicherheit (LAVES).

### Whole-genome sequencing

Genomic DNA was prepared using a DNeasy Kit (Qiagen) according to the manufacturer’s instructions and subjected to whole-genome sequencing by Macrogen (Macrogen Korea, Seoul, South Korea). Briefly, a 100 bp paired-end read library was prepared and sequenced on an Illumina HiSeq4000 (Illumina, San Diego, USA), which generated raw images utilizing HCS (HiSeq Control Software v3.3) for system control and base calling through the integrated primary analysis software RTA (Real Time Analysis v2.5.2). The BCL binary files were converted into FASTQ using the illumine package bcl2fastq2 (v2.16.0.10). Reads were aligned to a mouse reference genome (GRCm38.p4) with Burrows-Wheeler Aligner (http://bio-bwa.sourceforge.net/), duplicates were identified and removed using Picard software (http://broadinstitute.github.io/picard/), and variant information was found using SAMTools (http://samtools.sourceforge.net/). Deep sequencing yielded 514.93 million mapped reads corresponding to 27.1-fold average coverage (see Supplementary Table 1 for a complete list of variants).

### Preparation of lymphocytes from tissues

Secondary lymphoid organs (spleen, lymph nodes, or thymus) were homogenized through a 70 μm filter and washed with FACS buffer (PBS containing 3% fetal bovine serum (FBS)). Erythrocytes were removed by resuspension in ACK lysis buffer (0.15 M NH_4_Cl, 1 mM KHCO_3_, and 0.1 mM EDTA) for 5 min, followed by washing in FACS buffer. Euthanized mice underwent cardiac perfusion with 20 ml ice-cold PBS with heparin (100 U/ml). Lung tissue was cut into <1 mm fragments and then digested with 1 mg/ml collagenase D (Roche) at 37 °C for 60 min. The cells were then filtered through a 70 μm filter. The liver was minced and homogenized through a 70 μm filter. Lymphocytes were obtained from homogenates by histopaque (Sigma) density separation.

### Antibodies, cell staining, and flow cytometry

Staining for cell-surface markers was performed in FACS buffer for 20 min at 4 °C using the following antibodies: APC-Cy7-conjugated anti-CD8 (53-6.7), BV785-conjugated anti-CD4 (GK1.5), PerCP-Cy5.5-conjugated anti-CD4 (RM4-5), Pacific Blue-conjugated anti-CD3 (17A2), APC-Cy7-conjugated anti-CD3 (145-2C11), APC-conjugated anti-CD103 (2E7), BV421-conjugated anti-CD127 (IL-7Rα) (A7R34), APC-conjugated anti-CTLA-4 (UC10-4B9), APC-Cy7- or BV605-conjugated anti-CD62L (Mel-14), PE-conjugated anti-IFN-γ (XMG1.2), AF647-conjugated anti-IL-4 (11B11), and streptavidin-Cy5 (all from BioLegend); PE-conjugated anti-CD25 (PC61.5), Pacific Blue- or FITC-conjugated anti-CD25 (PC61 5.3), APC-conjugated anti-GITR (DTA-1), PE-conjugated anti-ICOS (7E.17G9), PE-Cy7-conjugated anti-PD-1 (543), FITC-, PE- or APC-conjugated anti-Foxp3 (FJK-16s), APC- or eF450-conjugated anti-CD44 (IM7), APC-conjugated anti-CCR7 (4B12), APC-conjugated anti-CXCR3 (CXCR3-173), biotinylated anti-CD29 (eBioHMb1-1), anti-CD18 (M18/2), anti-β7 (FIB504), anti-αv (RMV-7), and PE-Cy7-conjugated anti-IL-17A (eBio17B17) (all from Thermo Fisher Scientific); PE-conjugated anti-Thy1.1 (CD90.1) (OX-7) and AF647-conjugated anti-pStat5 (pY694) (clone 47) (from BD Biosciences); and Cy5-conjugated anti-Thy1.2 (CD90.2) (MMT 1; made in-house). For intracellular staining, fixation and permeabilization were performed according to manufacturer recommendations. Cryosections were stained with PE-conjugated anti-CD45.2 (104), Cy5-conjugated anti-CD3 (17A2, made in-house), Cy5-conjugated anti-B220 (TIB146, made in-house), APC-conjugated anti-TCRβ (H57-597, BioLegend), GFP Booster (ATTO488, Chromotek) and DAPI. For intracellular cytokine staining, cells were treated with 50 ng/ml PMA, 1.5 μg/ml ionomycin, and 10 μg/ml brefeldin A for 3 h and then fixed and permeabilized with the Intracellular Fixation & Permeabilization Buffer Set (eBioscience). To assess STAT5 phosphorylation, sorted GFP^+^ Treg cells were cultured in complete RPMI medium for 2 h at 37 °C and then stimulated with different concentrations of recombinant hIL-2 (Sigma) for 20 min, followed by staining using Phosflow Lyse/Fix Buffer and Phosflow Perm Buffer III (BD Biosciences). Simultaneous staining of surface and intracellular CD25 was performed using the same antibody labeled with different fluorochromes (clone PC61 5.3) together with an anti-Foxp3 antibody (FJK-16s) and the Foxp3/Transcription Factor Staining Buffer Set (eBioscience). Flow cytometry was performed on an LSR II, and data were analyzed using FlowJo software, while cell sorting was performed on a FACSAria IIu or a FACSAria Fusion (all BD Biosciences).

### T-cell expansion and an in vitro suppression assay

GFP^+^ Treg cells were sorted from pooled LNs and spleens isolated from Foxp3^GFP^ mice and cultured in RPMI 1640 medium (Gibco) supplemented with 10% heat-inactivated FBS (GE Healthcare Life Sciences), 2 mM l-glutamine, 1% penicillin-streptomycin (both from Gibco), and 50 μM β-mercaptoethanol (Sigma-Aldrich) at 37 °C with 5% CO_2_. For in vitro expansion, Treg cells were cultured in 96-well plates coated with 1 μg/ml anti-CD3 antibody (clone 17A2, prepared in-house) and 1 μg/ml anti-CD28 antibody (clone 37.51, eBioscience) in RPMI medium supplemented with 300 U/ml hIL-2 (Sigma) for 14 days. The cells were refed with fresh medium every second day. Total CD4^+^ T cells were isolated using the MACS CD4 Negative Isolation Kit II (Miltenyi Biotec) and labeled with the cell proliferation dye eFluor^TM^ 670 (eBioscience) according to the manufacturer’s instructions. To assess the suppressive capacity of Tregs in vitro, total CD4^+^ cells were stimulated with Dynabeads^TM^ Mouse T-Activator CD3/CD28 (Thermo Fisher) in the presence of WT or *Cd25*^Y129H^ Treg cells for 72 h.

### In vitro IL-2 capture assay

Sorted GFP^+^ Treg cells were cultured in 96-well U-bottom plates (5 × 10^5^/well) in 50 μl complete RPMI medium supplemented with recombinant hIL-2 (1.6 U/ml) for 3 h at 37 °C. The remaining IL-2 in the supernatant was detected using a cytometric bead array and the Human IL-2 Enhanced Sensitivity Flex Set (BD Biosciences) according to the manufacturer’s instructions.

### Intracellular cytokine production

Conventional CD4^+^ T cells (GFP^-^CD4^+^CD62L^+^CD44^−^CD25^−^) were sorted from WT or *Cd25*^Y129H^ Foxp3^GFP^ reporter mice and activated with plate-bound anti-CD3 (1 μg/ml) and anti-CD28 (1 μg/ml) antibodies in medium for 5 days under Th1 conditions (10 ng/ml IL-12, PeproTech; 50 U/ml IL-2, Sigma-Aldrich; 10 μg/ml anti-IL-4 mAb, BioLegend), Th2 conditions (20 ng/ml IL-4, PeproTech; 50 U/ml IL-2; 10 μg/ml anti-IFN-γ mAb, BioLegend), or Th17 conditions (20 ng/ml IL-6, PeproTech; 2 ng/ml TGF-β1, R&D Systems; 10 ng/ml IL-1β, PeproTech; 10 ng/ml IL-23, R&D Systems; 10 ng/ml anti-IFN-γ mAb; 10 μg/ml anti-IL-4 mAb). The medium was replaced after 3 days, and the cells were incubated for an additional 2 days before quantification.

### Vector construction and transfection

*Cd25* cDNA from WT and CD25^Y129H^ mice was amplified by using the following primers: forward 5′-CATCATGGATCCATGGAGCCACGCTTGCTGATGT-3′ and reverse 5′-CATCATGAATTCGATGGTTCTTCTGCTCTTCC-3′, which inserted BamHI and EcoRI restriction sites into the 5′ and 3′ ends, respectively. The PCR product was digested with BamHI and EcoRI restriction enzymes and subcloned into pcDNA3-mRFP (Addgene plasmid #13032). A total of 5 × 10^6^ 293T cells per 10-cm dish were seeded and transfected the next day using a calcium phosphate transfection kit (Sigma) with 10 μg plasmid. Transfected cells were cultured for 24 h in complete DMEM supplemented with 20 mM HEPES (Sigma-Aldrich) at 37 °C with 5% CO_2_ before FACS analysis.

### Induction of oral tolerance and assessment of delayed-type hypersensitivity (DTH) responses

To induce oral tolerance, mice were fed 25 mg ovalbumin (OVA) (grade III; Sigma-Aldrich) dissolved in 200 μl PBS or only PBS as a control by oral gavage on days 0 and 2. On day 7, the mice were immunized by subcutaneous injection of 300 μg OVA (grade VI; Sigma-Aldrich) in 200 μl PBS/CFA emulsion (containing 100 μg *Mycobacterium tuberculosis*; Sigma-Aldrich). On day 21, the mice were challenged by intradermal injection of 50 μg OVA (grade VI; Sigma-Aldrich) in 10 μl PBS into the right ear pinna, while 10 μl PBS without OVA was injected into the left ear pinna as a control. Ear swelling was then measured in a blinded fashion before and 48 h after injection by using a micrometer. OVA-specific ear swelling was calculated as follows: (right ear thickness − left ear thickness)_48h_ − (right ear thickness − left ear thickness)_0h_.

### Adoptive transfer colitis model

Colitis was induced in *Rag2*^−/−^ mice by intraperitoneal injection of 5 × 10^5^ FACS-sorted naive CD4^+^CD62L^+^CD44^−^CD25^−^ cells isolated from pooled spleens and lymph nodes from B6 (Thy1.2^+^) mice with or without 5 × 10^5^ sorted CD4^+^GFP^+^ Thy1.1^+^ Treg cells isolated from pooled spleens and lymph nodes from WT or CD25^Y129H^ Foxp3^GFP^ mice. Mice were monitored for weight loss and analyzed 8 weeks after cell transfer or immediately upon loss of more than or equal to 20% of their initial body weight.

### Histopathological analysis of colon samples

Colon samples were removed, fixed in neutral buffered 4% PFA, dehydrated (Shandon Hypercenter, XP), and subsequently embedded in paraffin (TES, Medite). Sections (2–3 μm thick, Reichert-Jung 2030 microtome) were deparaffinized in xylene and H&E stained according to standard protocols. Histological scoring was performed in a blinded manner as described previously.^23^

### RNA expression analysis

Total RNA was isolated from cells using an RNeasy Kit (Qiagen) or from PFA-fixed, paraffin-embedded colon tissue sections (6 μm sections; four sections per sample) using an RNeasy FFPE kit (Qiagen) according to the manufacturer’s instructions. Equivalent quantities of total RNA were reverse transcribed with SuperScript III reverse transcriptase (Invitrogen) and random hexamers according to the manufacturer’s protocol. Gene expression levels were determined by quantitative real-time PCR using SYBR Green PCR Master Mix (Takara) on an ABI 5700 sequence detector system (Applied Biosystems) using the following primers specifically designed to span intron/exon boundaries: CD25, 5′-GGGAAAACGGGGTGGACTC-3′ and 5′-CTGTGGTGGTTATGGGGCAG-3′; IL-17, 5′-ACTACCTCAACCGTTCCACG-3′ and 5′-GAGCTTCCCAGATCACAGAGG-3′; IFN-γ, 5′-AGCAAGGCGAAAAAGGATGC-3′ and 5′-TCATTGAATGCTTGGCGCTG-3′; TGF-β, 5′-CTGCTGACCCCCACTGATAC-3′ and 5′-AGCCCTGTATTCCGTCTCCT-3′; TNF-α, 5′-TTCTATGGCCCAGACCCTCA-3′ and 5′-GTTTGCTACGACGTGGGCTA-3′; and HPRT, 5′-TCCTCCTCAGACCGCTTTT-3′ and 5′-CCTGGTTCATCATCGCTAATC-3′. Gene expression levels were normalized to the expression of the housekeeping gene *Hprt*.

### Immunohistology

Sacrificed mice were perfused with 20 ml ice-cold PBS with heparin (100 U/ml). The salivary gland, lacrimal gland, and pancreas were removed, embedded in optimum cutting temperature (OCT) compound, and frozen on dry ice. Eight-micrometer sections were prepared with a cryostat (CM3050 Leica) and fixed for 10 min in ice-cold acetone. Imaging of tissue sections was performed using an automated slide scanner (AxioScan Z1; plan-apochromat objective: 10×/0.45 M27) equipped with an Axiocam 506 mono camera and ZenBlue analysis software (all from Zeiss). Organs were collected and fixed in 2% PFA/30% sucrose overnight (thymus and spleen) or for 1.5 h (LNs) before they were embedded in OCT compound and snap-frozen. Cryosections (7 μm in thickness) were blocked with 4% BSA, permeabilized with 0.5% Triton X-100 for 20 min, and stained with conjugated antibodies and DAPI (Sigma). Slides were mounted with MOWIOL (homemade), and images were acquired using a Zeiss Axiovert 200M microscope with a 20× objective. Cytospins of stained cells were performed by centrifugation of 100 μl cell suspension (5 × 10^5^ cells/ml PBS) at 450 rpm for 5 min in a Shandon Cytospin 4 (Thermo Scientific). Images were acquired with a Leica SP2 confocal microscope with a 63× Plan-Apochromat objective and analyzed using Imaris scientific software (Bitplane).

### DNA methylation analysis

Genomic DNA was isolated from sorted GFP^+^ Treg cells and GFP^-^ CD4^+^ T cells derived from WT or *Cd25*^Y129H^ Foxp3^GFP^ mice using a DNeasy kit (Qiagen) and concentrated using the DNA Clean & Concentrator Kit (Zymo Research), both following the manufacturer’s instructions. Methylation analysis of Treg cell-specific demethylated regions (TSDRs) was performed using bisulfite sequencing as described previously.^24^ We used the primers mTSDR-for (5′-bio-TAAGGGGGTTTTAATATTTATGAGGTTT-3′), mTSDR-rev (5′-CTAAACTAACCAACCAACTTCCTA-3′), mTSDR seq1 (5′-ACCCAAATAAAATAATATAAATACT-3′), mTSDR seq2 (5′-ATCTACCCCACAAATTT-3′), and mTSDR seq3 (5′-AACCAAATTTTTCTACCATT-3′) for standard amplification and pyrosequencing, whereas we used the primers mTSDR-sen-for (5′-AGGTTGTTTTTGGGATATAGAATATG-3′), mTSDR-sen-rev (5′-ACCTATAAAATAAATTATCTACCCCCTTC-3′), and mTSDR-sen-seq1 (5′-GTTGTTATAATTTGAATTTGGTTAG-3′) for low-input samples. Exclusively, cells from male mice were used for the methylation analysis. For all samples analyzed, cells from two mice per condition were pooled to collect a sufficient amount of DNA for methylation analysis.

### Single-cell sequencing of cell-hashed Treg cells and data preprocessing

CD4^+^GFP^+^ Treg cells were FACS sorted from the spleen of WT or CD25^Y129H^ Foxp3^GFP^ mice and labeled with TotalSeq cell hashing antibodies (BioLegend) according to the cell hashing protocol (https://cite-seq.com/protocol/; PMID: 30567574). After labeling, the cells from three mice per genotype were pooled and used to generate single-cell gel beads in emulsions using the Chromium Single Cell 3′ Library & Gel Bead Kit v.3 (10x Genomics) according to the manufacturer’s protocol (https://support.10xgenomics.com/single-cell-gene-expression/library-prep/doc/user-guide-chromium-single-cell-3-reagent-kits-user-guide-v3-chemistry). During reverse transcription, 1 µl HTO PCR additive primer (0.2 µM) was added to the cDNA amplification mixture to increase the yield of HTO products. After cDNA amplification, HTO-derived cDNAs were separated from mRNA-derived cDNAs using SPRI selection and further purified, amplified, and fragmented according to the cell hashing protocol or Chromium Single Cell 3′ Reagent Kit v3 user guide (10x Genomics), respectively. For sequencing, sample indexes were added during index PCR (12 cycles), and purified libraries were sequenced on a NextSeq 550 (Illumina) with 26 cycles of read 1, 8 cycles of i7 index, and 56 cycles of read 2. For cDNA libraries, Cell Ranger v3.0.2 was used for alignment to a mouse genome (mm10), filtering, barcode counting, and unique molecular identifier counting. This setup allowed us to confidently map 82% of 270,993,014 or 311,497,445 reads from WT or CD25^Y129H^ CD4^+^GFP^+^ Tregs, respectively, to the genome. Downstream analysis was performed using the Seurat R package (version 3.0, Satija Lab; PMID: 31178118), which enables the integrated processing of multimodal (RNA and HTO) single-cell datasets (PMID: 29608179). Initially, raw cDNA library datasets were loaded, and genes detected in less than three barcodes or barcodes with less than 200 genes were filtered out. Barcodes passing this initial filtering served for retrieval of the cell hashing tags that were counted using the CITE-seq-Count protocol (https://cite-seq.com/protocol/; PMID: 30567574), resulting in an average of 1111 and 967 reads/cell for WT and CD25^Y129H^ CD4^+^GFP^+^ Treg cells, respectively. Next, all barcodes designated “singlets” were extracted and further filtered using Seurat’s standard preprocessing workflow. Cells with low-quality transcriptomes (<900 detected genes), doublets (>3500 genes), and a percentage of mitochondrial genes >10% were removed from the analysis, resulting in a total of 3704 WT CD4^+^GFP^+^ Treg cells (with an average of 1702 genes/4722 reads) and 2142 CD25^Y129H^ CD4^+^GFP^+^ Treg cells (with an average of 1725 genes/4862 reads) passing the filters.

### Subpopulation detection in single-cell sequencing data and differential expression analysis

Unsupervised clustering and uniform manifold approximation and projection (UMAP) plots were generated using a resolution of 0.5 and the first 30 principal component analysis (PCA) dimensions by applying the SCTransform function of the Seurat package. After inspection, we detected the presence of a few contaminating B cells (Ms4a1^+^Cd3e^−^) that were excluded from further analyses. The remaining events were reclustered with a resolution of 0.5 and the first 30 PCA dimensions using the SCTransform function of the Seurat package, resulting in six clusters. Data were log normalized and scaled using the NormalizeData and ScaleData functions of Seurat, and differential expression testing was performed using the FindAllMarker or FindMarker function of Seurat to generate a list of DEGs. All neighboring clusters with fewer than 20 DEGs (adjusted *p* value < 0.05) were merged, resulting in a final UMAP plot with four clusters. Heatmaps and Venn diagrams for the DEGs were generated in R using the ggplot2 package. To identify enriched Gene Ontology (GO) biological processes, data were exported from R and analyzed with PANTHER classification system software (v.14.1) using Fisher’s exact test with a false discovery rate (FDR) calculated to be <0.05 and a significance level adjusted to 0.05 (http://pantherdb.org; PMID 23193289). To identify subset-specific markers, we manually compared the DEGs from each cluster, determined by a threshold for the FDR < 0.001, to markers previously reported in canonical pathways and Treg cell subsets using predefined^25^ and hallmark gene sets (https://www.gsea-msigdb.org/gsea/msigdb). The full set of signature genes is provided in Supplementary Table 2. The log_2_ fold change was calculated as log_2_(*B*) − log_2_(*A*), where *B* and *A* are the proportions of Treg cells in each cluster in *Cd25*^Y129H^ and WT mice, respectively.

### Pseudotime analysis of Treg cell differentiation

For trajectory analysis, we used the open-source R package Slingshot (available from the GitHub repository https://github.com/kstreet13/slingshot).^26^ Cells were ordered in pseudotime using PCA and Seurat cluster assignments. With cluster 2 (central Treg (cTreg) cells) chosen as the starting point, two main trajectories were found: one including cells from clusters 2, 3, and 4 and the other including cells from clusters 2, 3, and 1.

### Statistics

Statistical analysis was performed with Prism 8 (GraphPad Software). When comparing two groups, statistical significance was determined using a two-tailed unpaired *t* test. To compare multiple groups, one-way or two-way ANOVA was used. Statistical details, including the statistical test used and the number of mice analyzed (*n*), can be found in the legend of each figure. *p* values < 0.05 were considered statistically significant; data with *p* > 0.05 were considered to be not significant (ns). The following annotations were used: *p < 0.05, **p < 0.01, ***p < 0.001, and ****p < 0.0001. All data are presented as the mean ± SD.

## Results

### Characterization of Treg cells with a hypomorphic mutation in the *Cd25* gene

**Fig. 1:**
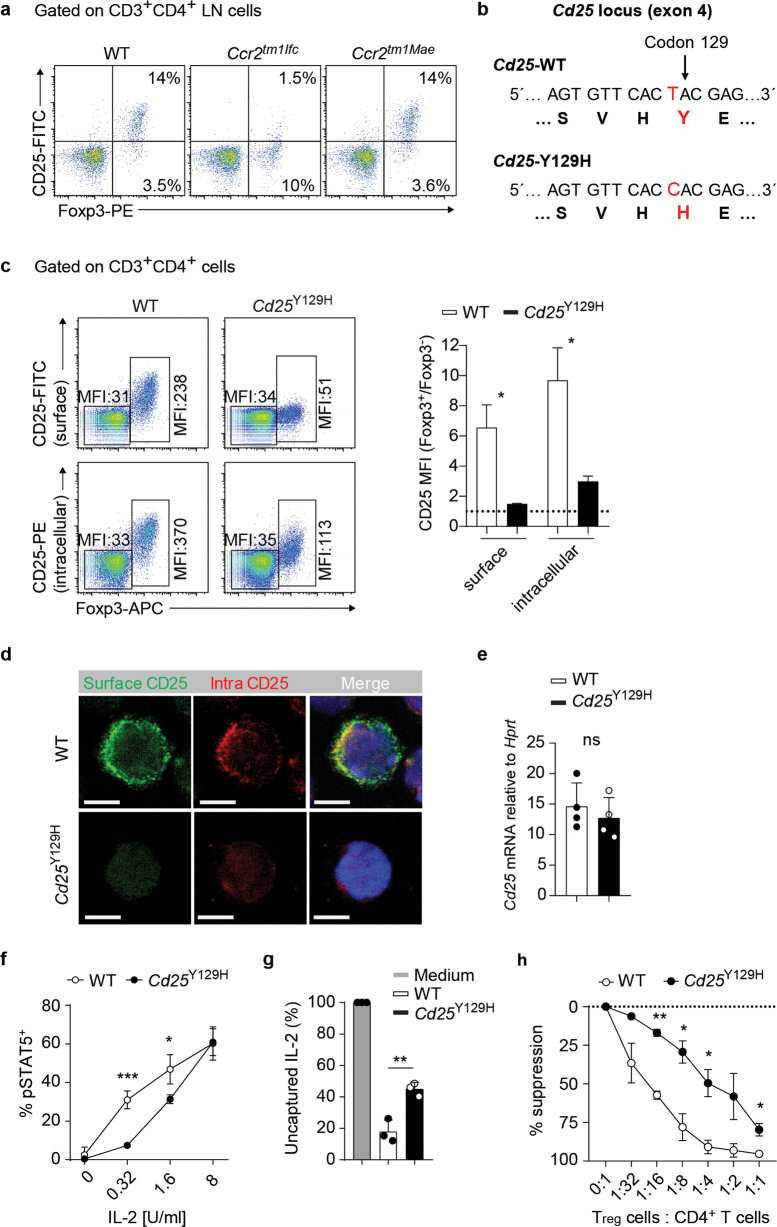
Identification and functional characterization of the hypomorphic *Cd25*^Y129H^ mutation. **a** FACS plots of CD3^+^CD4^+^ lymph node cells from wild-type (left), *Ccr2*^*tm1Ifc*^ (middle), and *Ccr2*^*tm1Mae*^ (right) mice after staining with anti-CD25 and anti-Foxp3 antibodies. Numbers within the dot plots indicate the percentage of cells within the gated region. Representative dot plots from two independent experiments with 3 mice per genotype are shown. **b** Nucleotide and encoded amino acid sequences of the *Cd25* locus in WT and *Cd25*^Y129H^ mice. **c** Expression of surface and intracellular CD25 by Foxp3^+^ Treg cells from WT and *Cd25*^Y129H^ mice analyzed by flow cytometry. Numbers in the plots indicate the mean fluorescence intensity (MFI) of CD25 and are summarized in the bar graph (right). Data were derived from two independent experiments with 2 mice per genotype. **d** Confocal microscopy images of WT and *Cd25*^Y129H^ Treg cells; surface CD25—green; intracellular CD25—red; DAPI—blue. Images are representative of two independent experiments; scale bar 5 μm. **e** qRT-PCR analysis of *Cd25* mRNA expression in Foxp3^+^ Treg cells from WT and *Cd25*^Y129H^ mice. Data are derived from four independent experiments with 4 mice per genotype analyzed. **f** Intracellular tyrosine-phosphorylated STAT5 (pSTAT5) in WT (open circles) and *Cd25*^Y129H^ (filled circles) Foxp3^+^ Treg cells stimulated in vitro for 20 min with different amounts of IL-2. Data were derived from three independent experiments. **g** Bead-based cytometric analysis of IL-2 in the medium 3 h after culture without (gray bar) or with Foxp3^+^ Tregs from WT (white bar) or *Cd25*^Y129H^ (black bar) mice. Data were derived from three independent experiments. **h** Comparison of the ability of WT (open circles) and *Cd25*^Y129H^ (filled circles) Foxp3^+^ Treg cells to suppress CD4^+^ T-cell proliferation in vitro. Serial numbers of Treg cells were cultured with ef670-labeled total CD4^+^ T cells for 3 days with beads coated with anti-CD3 and anti-CD28 antibodies. The proliferation of CD4^+^ T cells was determined by flow cytometry, and the results were calculated as the percentage of suppression. Data were derived from two independent experiments and are represented as the mean ± SD; ns not significant; *p < 0.05, **p < 0.01, ***p < 0.001; two-tailed unpaired Student’s *t* test

During routine flow cytometric analysis, we observed reduced CD25 expression in Treg cells and defined thymocyte subpopulations, as well as in in vitro activated CD4^+^ T cells from one *Ccr2*-deficient (*Ccr2*^*tm1Ifc*^) strain but not from another *Ccr2-*deficient (*Ccr2*^*tm1Mae*^) strain (Fig. 1a and Supplementary Fig. 1). We performed whole-genome DNA sequencing to determine whether a spontaneous mutation in *Ccr2*^*tm1Ifc*^ mice could be responsible for the low CD25 expression. From all the sequence variants found, we identified one affecting the *Cd25* gene (Supplementary Table 1). The identified variant carried a point mutation (T–C) at position Chr2:11680290 in exon 4 that resulted in the replacement of tyrosine (Y) with histidine (H) at codon 129 (Y129H) (Fig. 1b). Based on the known three-dimensional structure of human CD25,^27^ Y129 is located in the extracellular part of the protein and probably forms a hydrogen bond with P8 that may help to stabilize a region of the CD25 protein that is in direct contact with IL-2 (Supplementary Fig. 2a). The presence of the Y129H mutation was confirmed independently by Sanger sequencing of the genomic DNA fragment in *Ccr2*^*tm1fFc*^ mice (data not shown). Moreover, to avoid confounding effects, the mutated *Ccr2* alleles were replaced by backcrossing to B6 mice for at least three generations, leading to the generation of *Cd25*^wt/Y129H^ mice. Intercrossing these heterozygous mutants generated homozygous mutant mice (*Cd25*^Y129H^) that were used for all experiments together with their WT littermates as controls. Homozygous *Cd25*^Y129H^ mice were fertile and survived until adulthood with no obvious developmental defects (data not shown). To better understand the molecular mechanism by which the *Cd25*-Y129H mutation results in decreased CD25 expression, we analyzed the protein and mRNA levels of CD25 by Foxp3^+^ Treg cells from WT and *Cd25*^Y129H^ mice. The mean fluorescence intensity (MFI) of both cell surface and intracellular CD25 was strongly diminished in *Cd25*^Y129H^ mice (Fig. 1c). In addition, we found similar results in in vitro activated CD4^+^ T cells from WT and *Cd25*^Y129H^ mice and in 293T cells transiently transfected with plasmids encoding the CD25-WT or CD25-Y129H gene (Supplementary Fig. 3a, b). This result was confirmed by confocal immunofluorescence microscopy, which showed that CD25 downregulation was especially prominent at the cell-surface level, whereas the intracellular CD25 expression levels were better sustained (Fig. 1d). Moreover, quantification of *Cd25* transcript levels by real-time PCR revealed that WT and *Cd25*^Y129H^ Treg cells expressed comparable amounts of *Cd25* mRNA, rendering the possibility that the *Cd25*-Y129H mutation affects mRNA transcription or stability unlikely (Fig. 1e). However, following in vitro stimulation of CD4^+^ T cells with anti-CD3 and anti-CD28 mAbs, we found reduced levels of the *Cd25* transcript in *Cd25*^Y129H^ cells compared to WT cells, suggesting that transcriptional activity might be affected in mutant *Cd25*^Y129H^ T cells subsequent to activation via the TCR (Supplementary Fig. 3c). Alternatively, reduced CD25 expression could restrict the IL-2/CD25 positive feedback loop, resulting in decreased mRNA synthesis. Moreover, we also showed that the CD25 protein does not accumulate intracellularly, excluding a hypothetical defect in protein transport to the cell membrane in *Cd25*^Y129H^ cells. Irrespective of the underlying course, our data indicated that the *Cd25*^Y129H^ mutation led to reduced levels of CD25.

To demonstrate a functional consequence of the diminished expression of CD25 in *Cd25*^Y129H^ Treg cells, we assessed IL-2-induced phosphorylation of signal transducer and activator of transcription 5 (pSTAT5), which reflects a main outcome of IL-2R signaling. WT and *Cd25*^Y129H^ Treg cells displayed different dose responses to recombinant hIL-2 (Fig. 1f). At a low IL-2 concentration (0.32 U/ml), the magnitude of the response (measured as % of pSTAT5^+^ cells) was significantly higher (fourfold) in WT Treg cells than in *Cd25*^Y129H^ Treg cells, whereas at a high IL-2 concentration (8 U/ml), the fraction of pSTAT5^+^ cells was comparable between WT and *Cd25*^Y129H^ Treg cells. These results show that the reactivity of *Cd25*^Y129H^ Treg cells to physiological amounts of IL-2, which usually do not exceed 0.05 U/ml, is diminished.^28^ We next asked whether *Cd25*^Y129H^ Treg cells can take up and deplete exogenous IL-2, which has been suggested to be an essential mechanism of Treg cell-mediated suppression.^11,29,30^ Using an in vitro assay, we found that IL-2 consumption by *Cd25*^Y129H^ Treg cells was significantly reduced compared to that by WT Treg cells (Fig. 1g). This finding suggests that in vivo, low CD25 expression by *Cd25*^Y129H^ Treg cells may decrease their capacity to compete for IL-2 produced by activated T cells, which in turn could potentially lead to a diminished capacity to suppress the proliferation of conventional T cells. Moreover, by comparing the in vitro suppressive activity of WT and *Cd25*^Y129H^ Treg cells by evaluating their ability to inhibit CD4^+^ T-cell proliferation at different cell ratios, we found that the suppressive capacity of *Cd25*^Y129H^ Treg cells was strongly reduced (Fig. 1h). Altogether, these results demonstrate that reduced expression of CD25 in *Cd25*^Y129H^ Treg cells significantly impairs their responsiveness to IL-2 and their immunosuppressive capacity.

### Treg cells in *Cd25*^Y129H^ mice exhibit altered homeostasis and phenotype

**Fig. 2:**
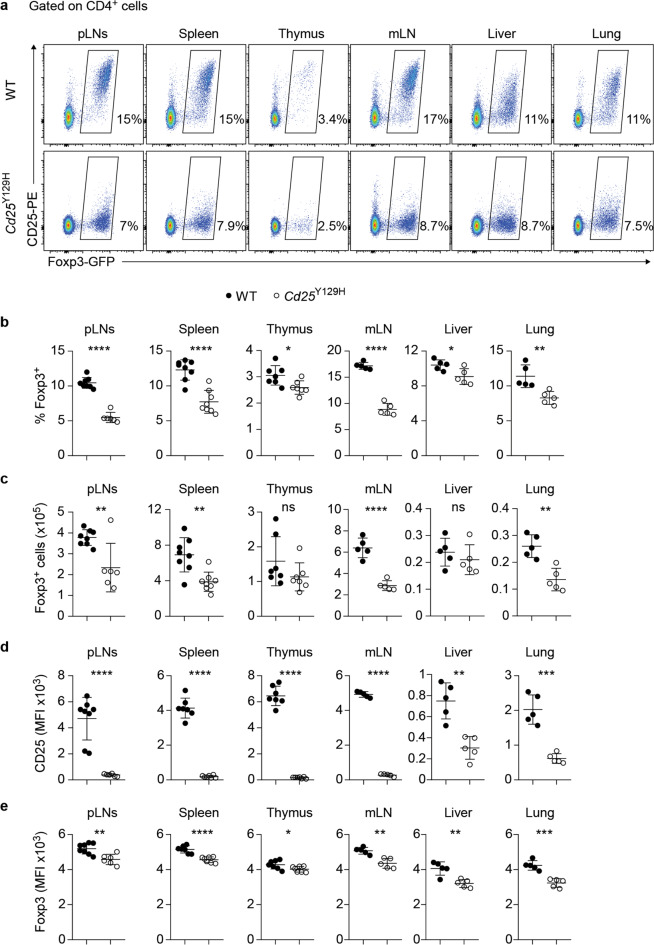
The hypomorphic *Cd25*^Y129H^ mutation leads to reduced CD25 expression and loss of Foxp3^+^ Treg cells. **a** Representative FACS plots showing the expression of Foxp3 and CD25 by CD4^+^ T cells in the peripheral lymph nodes (pLNs), spleen, thymus, mesenteric lymph nodes (mLNs), liver, and lungs of 8–12-week-old WT and *Cd25*^Y129H^ mice. **b** Percentages and **c** absolute cell numbers of Foxp3^+^ Treg cells in different organs of WT (filled circles) and *Cd25*^Y129H^ (open circles) mice. Mean fluorescence intensities (MFIs) of CD25 (**d**) and Foxp3 (**e**) on Treg cells from WT and *Cd25*^Y129H^ mice. Data were derived from at least three experiments with 5–8 mice per genotype and are represented as the mean ± SD. ns not significant; *p < 0.05, **p < 0.01, ***p < 0.001, ****p < 0.0001; two-tailed unpaired Student’s *t* test

Examination of thymocytes from WT and *Cd25*^Y129H^ mice revealed similar percentages and absolute cell numbers of CD4^−^CD8^−^ double-negative, CD4^+^CD8^+^ double-positive, and CD4^+^ and CD8^+^ single-positive thymocytes, suggesting that the *Cd25*^Y129H^ mutation does not impact thymocyte development (Supplementary Fig. 4a). Likewise, the percentages and absolute numbers of CD4^+^ T cells, CD8^+^ T cells, and CD44^hi^CD4^+^ and CD44^hi^CD8^+^ effector/memory T cells were also largely comparable between WT and *Cd25*^Y129H^ mice in peripheral lymphoid organs (lymph nodes and spleen), indicating that the *Cd25*^Y129H^ mutation does not substantially impair peripheral T-cell activation (Supplementary Fig. 4b–e). Next, we examined the Treg cell compartment in lymphoid organs and nonlymphoid tissues (NLTs) by assessing the expression of Foxp3 (Fig. 2a). Overall, the percentages and absolute cell numbers of Treg cells were significantly reduced in *Cd25*^Y129H^ mice compared to their WT littermates (Fig. 2b, c). Compared with that in WT mice, the percentage of Treg cells among CD4^+^ T cells in *Cd25*^Y129H^ mice was reduced by ~50% in the pLNs, spleen, and mLNs but was less pronounced in NLTs. Consistent with the smaller percentages, the absolute numbers of Treg cells were also significantly reduced in *Cd25*^Y129H^ mice. Interestingly, this trend was less pronounced in the thymus, where the percentages and absolute cell numbers of Treg cells among CD4SP thymocytes were only slightly lower in *Cd25*^Y129H^ mice than in WT mice (Fig. 2b, c). As expected, the expression of CD25 in Treg cells was strongly reduced in *Cd25*^Y129H^ mice (Fig. 2d). Notably, *Cd25*^Y129H^ Treg cells displayed a modest but significant reduction in Foxp3 expression, as indicated by the diminished MFI of Foxp3-GFP (Fig. 2e).

Next, we compared the expression of adhesion molecules and homing receptors between WT and *Cd25*^Y129H^ Treg cells from different lymphoid tissues and NLTs. Relative to WT Treg cells, *Cd25*^Y129H^ Treg cells showed upregulated expression of the chemokine receptor CXCR3 and several integrins and downregulated expression of the lymphoid homing chemokine receptor CCR7 (Supplementary Fig. 5a). In contrast, in non-Treg cells, these molecules were expressed at similar levels in both mouse strains (Supplementary Fig. 5b). This result suggested that in Treg cells, IL-2R signaling might confer a phenotype allowing them to home to lymphoid tissues rather than to NLTs. Despite showing an “effector” phenotype, *Cd25*^Y129H^ Treg cells were distributed similarly to their WT counterparts in different lymphoid organs, as revealed by histology (Supplementary Fig. 5c). Thus, our results suggested that efficient IL-2R signaling was required for full Treg cell homeostasis and phenotypic diversity maintenance.

### Enhanced activated/effector-like phenotype of *Cd25*^Y129H^ Treg cells

**Fig. 3:**
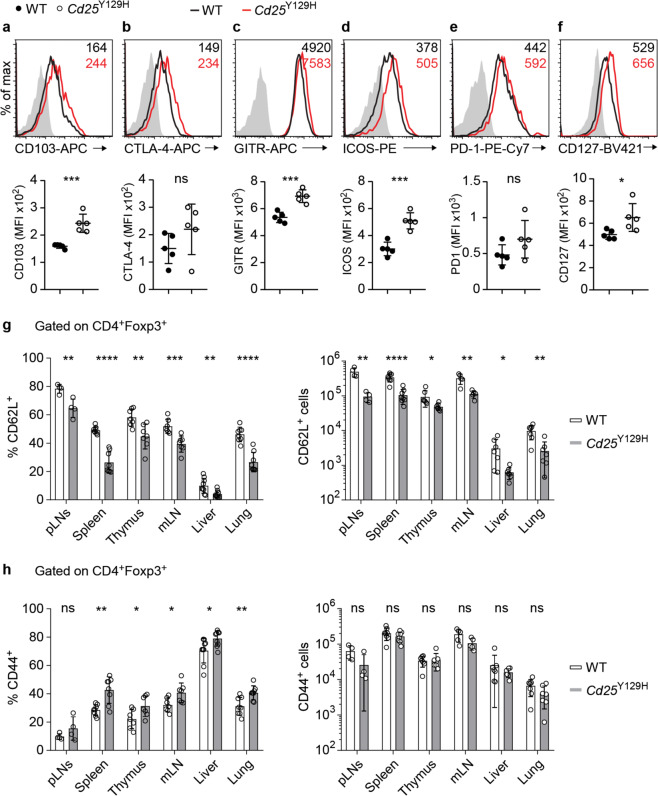
Activated/effector-like phenotype of Foxp3^+^ Treg cells in *Cd25*^Y129H^ mice. **a**–**f** Representative histograms (upper panels) and quantitative analysis (lower panels) of the MFIs of the indicated Treg surface markers on spleen CD4^+^Foxp3^+^ Treg cells from WT (filled circles) and *Cd25*^Y129H^ (open circles) mice. Data were derived from at least three independent experiments with 5 mice per genotype. Percentages (left) and absolute cell numbers (right) of CD62L^+^ (**g**) and CD44^+^ (**h**) Treg cell populations in different organs of WT and *Cd25*^Y129H^ mice. Data were derived from at least three independent experiments with 6–8 mice per genotype and are represented as the mean ± SD; ns not significant; *p < 0.05, **p < 0.01, ***p < 0.001; two-tailed unpaired Student’s *t* test

It has been reported that in the absence of IL-2R signaling, Treg cells show an activated phenotype.^4^ We therefore measured the levels of key Treg cell signature molecules in WT and *Cd25*^Y129H^ mice using flow cytometry. *Cd25*^Y129H^ Treg cells expressed higher levels of markers associated with cell activation, including CD103 (Fig. 3a), GITR (Fig. 3c), and ICOS (Fig. 3d), while the expression of markers associated with Treg cell suppressive function, including CTLA-4 (Fig. 3b) and PD-1 (Fig. 3e), was not significantly different from that of WT Treg cells. Furthermore, reduced expression of CD25 in *Cd25*^Y129H^ Treg cells was accompanied by increased expression of CD127 (IL-7Rα) (Fig. 3f). Similar results were found in both CD62L^+^CD44^−^ Treg cells and CD62L^−^CD44^+^ Treg cells (data not shown). However, *Cd25*^Y129H^ mice had reduced percentages and absolute cell numbers of CD62L^+^CD44^−^ Treg cells in lymphoid tissues and NLTs (Fig. 3g). Conversely, the absolute cell numbers of CD62L^−^CD44^+^ Treg cells were comparable between WT and *Cd25*^Y129H^ mice, although increased percentages of CD62L^−^CD44^+^ Treg cells were observed in *Cd25*^Y129H^ mice (Fig. 3h). Altogether, these results indicated that reduced expression of CD25 specifically impaired the maintenance of CD62L^+^CD44^−^ Treg cells, regardless of whether they resided in lymphoid tissues or NLTs, a finding that is consistent with previous reports showing that IL-2R signaling is specifically required for cTreg cell homeostasis.^31,32^

### Lack of spontaneous autoimmunity in *Cd25*^Y129H^ mice

**Fig. 4:**
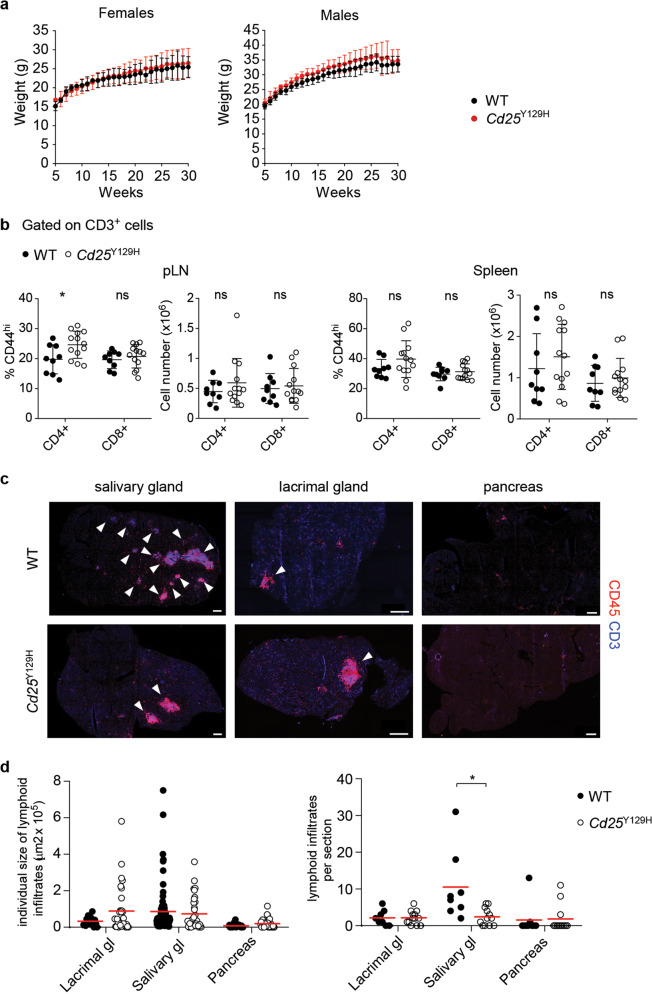
Lack of autoimmune symptoms in aged *Cd25*^Y129H^ mice. **a** Weekly body weight monitoring of female (left) and male (right) WT (black line) and *Cd25*^Y129H^ (red line) mice. Data were derived from 6 to 12 mice per genotype and sex. **b** Percentages and absolute cell numbers of CD44^hi^ cells among CD4^+^ and CD8^+^ T cells from the pLNs (left) and spleen (right) of WT (filled circles) and *Cd25*^Y129H^ (open circles) mice. Data were derived from at least three independent experiments with 9–13 mice per genotype. **c** Representative immunofluorescence microscopy of the salivary glands, lacrimal glands, and pancreas of WT (top) and *Cd25*^Y129H^ mice (bottom) stained with the indicated antibodies. Scale bar, 500 μm. **d** Individual size (left) and number (right) of lymphoid infiltrates per section in the salivary glands, lacrimal glands, and pancreas of WT (filled circles) and *Cd25*^Y129H^ (open circles) mice. Data were derived from at least three independent experiments with 9–13 mice per genotype. **a**–**d** Organs were isolated from 35- to 40-week-old mice. Data are shown as the mean ± SD. ns not significant; *p < 0.05 (two-tailed unpaired Student’s *t* test)

We next investigated whether *Cd25*^Y129H^ mice exhibited signs of autoimmunity, as previously reported for mice lacking expression of various IL-2R signaling components.^4,11–14^ Compared with WT mice, *Cd25*^Y129H^ mice showed a similar gain in body weight with age (Fig. 4a), had no substantial expansion of CD4^+^ or CD8^+^ T cells, and showed comparable percentages and absolute numbers of cells displaying a CD44^hi^ memory phenotype (Fig. 4b). In addition, histopathological analysis of aged mice (35–40 weeks old) of both strains revealed comparable lymphocyte infiltration in multiple organs, such as the salivary gland, lacrimal glands, and pancreas (Fig. 4c, d). Thus, the absence of autoimmunity in *Cd25*^Y129H^ mice likely indicates that Treg cells developing under this condition of impaired IL-2R signaling are still functional and maintain their suppressive capacity during aging. Alternatively, the lack of autoimmunity in *Cd25*^Y129H^ mice could also reflect a loss of IL-2R function in autoreactive CD4^+^ and CD8^+^ T cells. To explore this possibility, conventional CD4^+^ T cells from these mice were stimulated with anti-CD3/CD28 antibodies and subjected to T helper cell differentiation in vitro. Compared to WT cells, T cells from *Cd25*^Y129H^ mice exhibited significantly lower intracellular Th1 (IFN-γ) cytokine expression, similar Th2 (IL-4) cytokine expression, and modestly elevated Th17 (IL-17) cytokine expression (Supplementary Fig. 6). These data suggest that IL-2R signaling is required for the maximal generation of IFN-γ-producing cells during a type 1 immune response. Moreover, the abnormal responsiveness of potentially autoreactive T cells may partially contribute to the lack of spontaneous autoimmunity in *Cd25*^Y129H^ mice.

### IL-2R signaling balances the landscape of Treg cell subsets

**Fig. 5:**
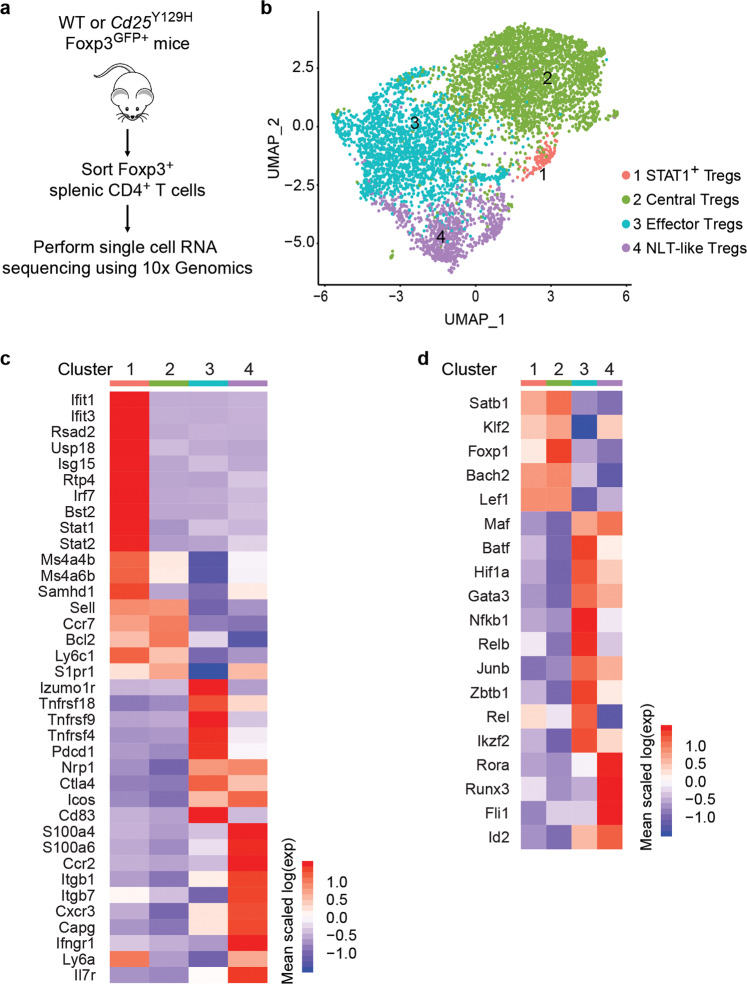
Identification of Treg cell subpopulations by single-cell RNA-seq analysis. **a** Experimental workflow for scRNA-seq analysis including sorting of splenic Foxp3^GFP^ Treg cells from WT or *Cd25*^Y129H^ mice and use of a Chromium Single Cell 3′ Reagent Kit. **b** UMAP-based clustering of merged scRNA-seq profiles of WT and *Cd25*^Y129H^ Treg cells. Heatmap showing the expression of selected marker genes (**c**) and selected transcription factors (**d**) for each of the four clusters shown in **b**. A complete list of the genes and expression values is available in Supplementary Table 3

To understand how IL-2R signaling shapes the heterogeneity of different Treg cell subsets at the molecular level, we performed scRNA sequencing of splenic CD4^+^Foxp3^+^ Treg cells from WT and *Cd25*^Y129H^ mice (Fig. 5a). Unsupervised graphical clustering organized the Treg cells into four clusters and identified transcripts that best characterized the different groups (Fig. 5b; Supplementary Table 3). To associate each cluster with known Treg cell subsets, we screened the 60 most significantly upregulated genes of each cluster and overlapped them with previously reported Treg cell markers and canonical signaling pathways (Fig. 5c; Supplementary Table 2). Cluster 1 highly expressed genes, such as *Ifit1, Ifit3, Isg15*, and *Stat1*, associated with type I interferon (IFN) responses but also genes that are primarily expressed by naive cells (e.g., *Sell* and *Ly6c1)* that most likely reflect an intermediate state of cell activation or direct response to IFN. We named this population Stat-1^+^ Treg cells, as suggested previously for Treg cells isolated from the brachial LNs.^33^ Cluster 2 included genes expressed in cTreg cells,^31,32^ such *as Sell, Ccr7, Bcl2, Ly6c1*, and *S1pr1*. Cluster 1 and cluster 2 Treg cells also showed increased expression of Ms4a4b and Ms4a6b, which may facilitate the resting state of these Treg cell subpopulations by inhibiting cell cycle progression.^34^ Cluster 3 was enriched for genes that were highly expressed in effector Treg (eTreg) cells.^35,36^ This cluster included key Treg cell signature genes, such as *Ctla4, Tnfrsf9* (4-1BB), and *Izumo1r*, as well as cell-surface receptors, such as *Tnfrsf4* (Ox40), *Tnfrsf18* (GITR), *Pdcd1* (PD-1), *Icos*, and *Nrp1*. Furthermore, the expression of many transcription factors that control the differentiation and function of eTreg cells,^37^ such as *Batf, Ikzf2* (Helios), *Maf*, and *Gata3*, was upregulated in cluster 3, whereas the expression of *Klf2, Satb1*, *Bach2*, and *Lef1*, which antagonize eTreg cell differentiation,^38–41^ was increased in cluster 2 (Fig. 5d). In addition, cluster 3 also overexpressed genes associated with the response to TCR signals, such as *Rel, Orai1*, and *Zap70*, which reflects recent engagement of the TCR. Some of the cell-surface markers that are characteristic of activated Treg cells, such as *Maf, Nrp1*, and *Icos*, were also highly expressed in cluster 4, consistent with the idea that these clusters identify Treg cell subpopulations that represent a continuum of discrete states rather than separate entities.^33^ This cluster also included transcription factors that promote the differentiation or function of tissue-resident Treg cells,^37^ such *as Rora, Runx3, Fli1*, and *Id2*, as well as genes that are functionally related to tissue homing and trafficking, including *Itgb1*, *Itgb7*, *Cxcr3*, and *Ccr2*, indicating that cluster 4 probably corresponds to cells undergoing a later stage of maturation and preadjustment to their trafficking to NLTs. We named this population NLT-like Treg cells, as previously described.^33^

**Fig. 6:**
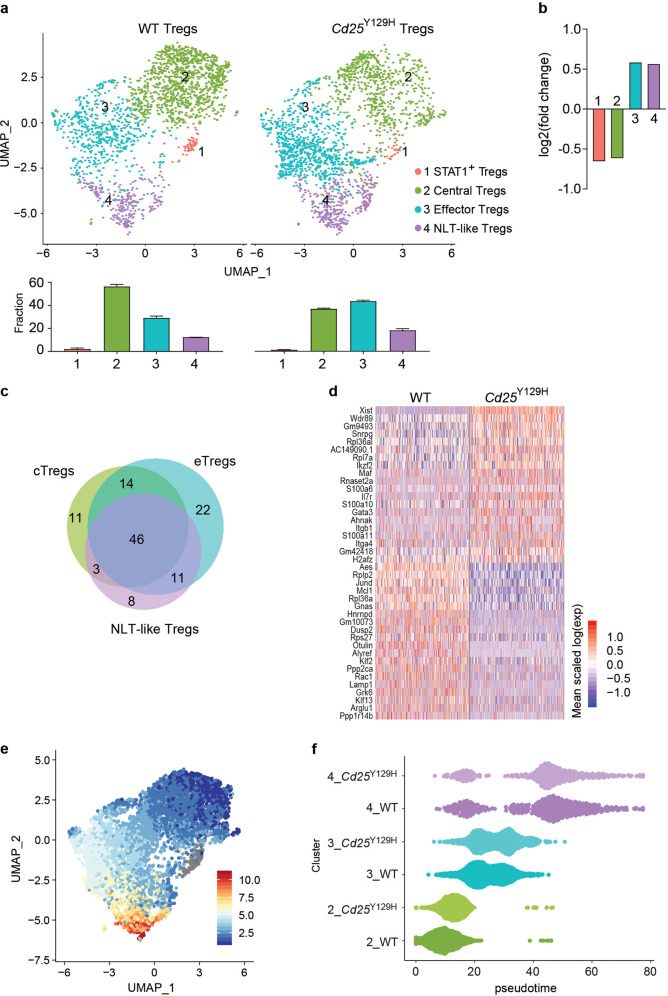
Comparison of WT and *Cd25*^Y129H^ Treg cell subpopulations by single-cell RNA-seq. UMAP-based clustering (**a**) and quantitative analysis (**b**) of splenic Treg cells from WT and *Cd25*^Y129H^ mice. Data were derived from 3 mice per group and are represented as log_2_ fold changes (see the “Materials and methods”). **c** Venn diagram showing the overlap among clusters 2, 3, and 4 for genes differentially expressed between WT and *Cd25*^Y129H^ Treg cells. **d** Heatmap showing the top 20 differentially expressed genes in WT and *Cd25*^Y129H^ Treg cells. A complete list of the genes and expression values is shown in Supplementary Table 5. **e**, **f** Trajectory inference analysis performed with Slingshot software

Next, we compared the proportion of each Treg cell subset between WT and *Cd25*^Y129H^ mice. Whereas the cTreg cell subset was significantly reduced, the eTreg cell subset was highly enriched in *Cd25*^Y129H^ mice (Fig. 6a, b). The Stat-1^+^ and NLT-like Treg cell subsets had similar abundances in both WT and *Cd25*^Y129H^ mice. These data were consistent with flow cytometry data showing that *Cd25*^Y129H^ Treg cells contained a higher fraction of CD44^+^CD62L^−^ Tregs (Fig. 3h). In addition, differential expression analysis revealed that over 40% of the DEGs in WT and *Cd25*^Y129H^ Treg cells largely overlapped among clusters 2, 3, and 4, indicating that IL-2R signaling is essential in maintaining the transcriptional programs of these different Treg cell subsets (Fig. 6c; Supplementary Table 4). This was in contrast with cluster 1 cells, which displayed very few DEGs between WT and *Cd25*^Y129H^ Treg cells and had reduced overlap with the other clusters, suggesting that the homeostasis of this cluster is largely IL-2R independent (Supplementary Fig. 7a). However, better surface markers must be identified to clarify this point. An overall comparison of the gene expression profiles of splenic WT and *Cd25*^Y129H^ Treg cells showed that 64 genes were upregulated and 29 genes were downregulated in WT Treg cells compared to *Cd25*^Y129H^ Treg cells (Fig. 6d; Supplementary Table 5). WT Treg cells expressed higher levels of genes required for T-cell maintenance, such as the antiapoptotic molecules *Mcl-1* and *Bclb11* (Supplementary Fig. 7b), consistent with a role of IL-2R signaling in T-cell survival.^42,43^ In contrast, *Cd25*^Y129H^ Treg cells showed higher expression of CD127, indicating that they may take advantage of IL-7 signaling for survival to compensate for reduced IL-2R signaling. In addition, the most significantly upregulated genes in WT Treg cells included many DNA-binding transcription regulators, such as *Klf2, Cited2, Pura, Crebzf, Jund*, and some transcriptional repressors, including *Aes* (aka Tle5), *Ncor1*, and *Suds3*, confirming that IL-2R signaling regulates the gene transcription of Treg cells.^44^ Next, GO-based enrichment analysis found that biological processes associated with metabolism, biosynthesis, and gene expression were significantly overrepresented in WT Treg cells (Supplementary Fig. 7c). In contrast, the genes upregulated in *Cd25*^Y129H^ Treg cells were associated with eTreg cell development and function, including *Ikzf2, Maf, Itgb1, Gata3, S100a6, S100a10*, and *S100a11*, as previously suggested^45^ (Fig. 6d). Interestingly, this list of genes strongly overlapped with the genes associated with cluster 4 (Supplementary Table 3), reinforcing the idea that IL-2R signaling balances Treg cell heterogeneity, and that decreased IL-2R signaling shifts the balance toward an effector phenotype. To further explore this hypothesis, we evaluated the Treg cell clusters with regard to their progression using the Slingshot software package.^26^ We found that the inferred spectrum of differentiation partially recapitulated cell clustering, ordering cTregs and eTregs followed by NLT-like Tregs on a linear developmental trajectory (Fig. 6e). Using the same approach, we also observed that *Cd25*^Y129H^ cTreg and eTreg cells had a developmental pseudotime tilted toward a more effector phenotype, indicating faster acquisition of effector properties under impaired IL-2R signaling (Fig. 6f). In accordance with recent studies, our scRNA-seq analysis identified the presence of different subpopulations of Treg cells in steady-state lymphoid tissues. In addition, by comparing the single-cell transcriptomes of Treg cells receiving optimal or low IL-2R signaling, we identified gene programs associated with Treg cells that depend on full IL-2 signaling. Altogether, these data demonstrate that impaired IL-2R signaling does not allow maintenance of the heterogeneity of Treg cells.

### Diminished oral tolerance in *Cd25*^Y129H^ mice

**Fig. 7:**
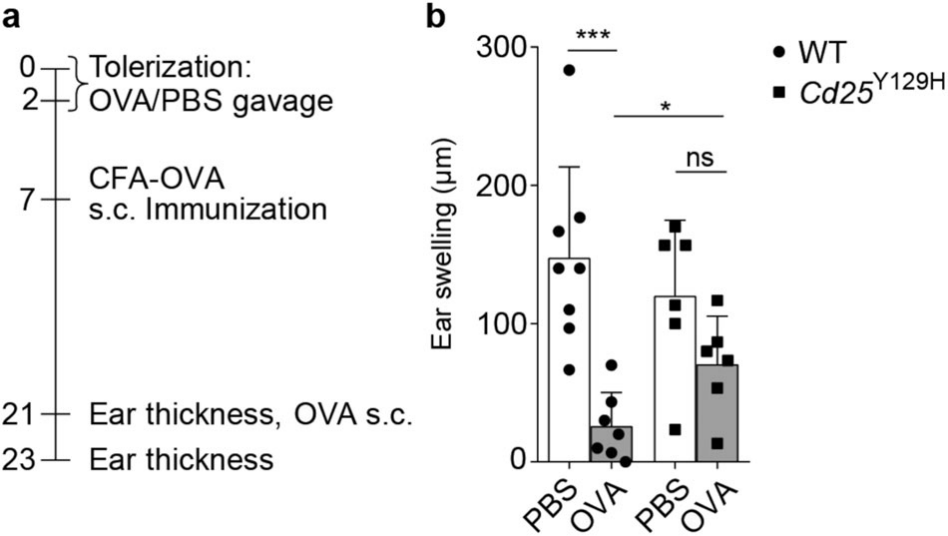
*Cd25*^Y129H^ mice failed to develop oral tolerance. **a** Flow chart of tolerance induction protocol. **b** WT (circles) and *Cd25*^Y129H^ (squares) mice were fed twice with 25 mg ovalbumin in PBS (OVA; gray bars) or PBS alone (white bars). On day 7, all mice were immunized by subcutaneous injection of OVA emulsified in CFA and challenged 2 weeks later with subcutaneous injection of OVA in PBS into the ear. Tolerance induction was analyzed by measuring ear swelling at 48 h after the challenge (see “Materials and methods”). Data were derived from at least three independent experiments with 6–8 mice per group and are represented as the mean ± SD. ns not significant; *p < 0.05, ***p < 0.001 (two-tailed unpaired Student’s *t* test)

Local expansion of Treg cells in the intestinal lamina propria has been shown to be essential for the establishment of tolerance to food antigens.^46^ Thus, we used *Cd25*^Y129H^ mice to examine the role of IL-2R signaling in the induction of oral tolerance in a DTH model. To this end, WT and *Cd25*^Y129H^ mice received either two doses of 25 mg OVA or PBS by oral gavage (Fig. 7a). One week later, the mice were sensitized by subcutaneous immunization with OVA/CFA. Two weeks later, induction of tolerance was assessed by measuring DTH reactions to intradermally injected OVA. WT mice fed OVA showed reduced DTH responses compared to mice fed PBS, indicating the induction of oral tolerance (Fig. 7b). In contrast, under the same conditions, *Cd25*^Y129H^ mice failed to develop oral tolerance, as shown by an impaired capacity to suppress DTH responses, suggesting a defective function of Treg cells in *Cd25*^Y129H^ mice.

### *Cd25*^Y129H^ Treg cells possess a reduced in vivo suppressive capacity

**Fig. 8:**
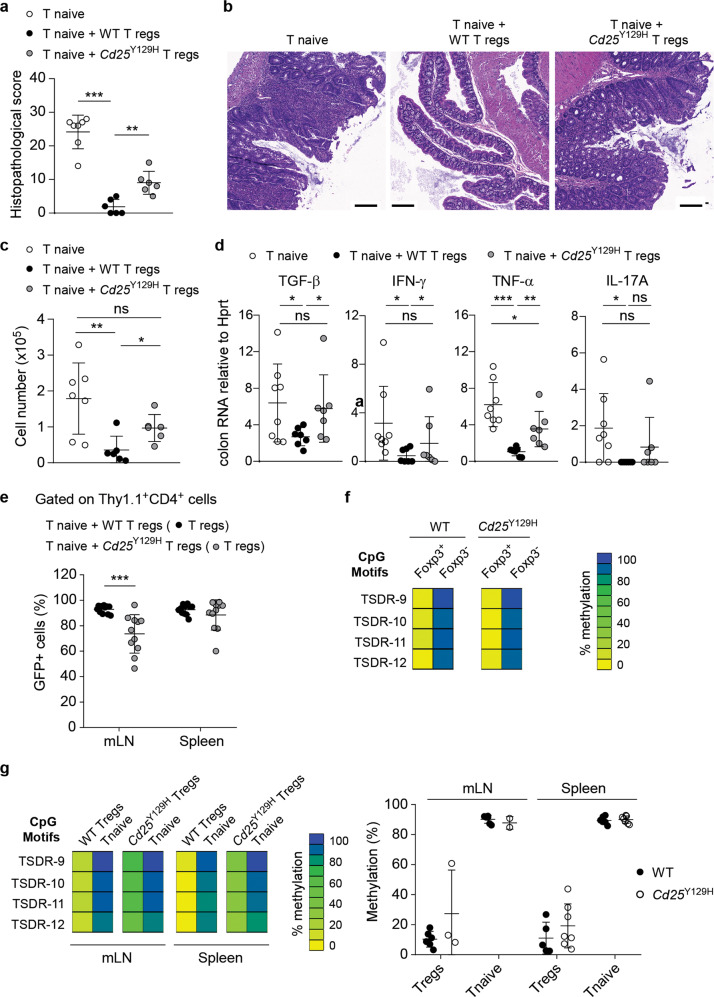
Treg cells from *Cd25*^Y129H^ donors show reduced in vivo suppressive function and lineage stability in a cell transfer model of colitis. **a** Histopathological scoring of colon tissue from Rag2^−/−^ mice 8 weeks after intraperitoneal transfer of naive T cells (CD25^−^CD62L^+^CD4^+^) alone (open circles) or with WT (filled circles) or *Cd25*^Y129H^ (gray circles) GFP^+^Foxp3^+^ Treg cells at a 1:1 ratio. **b** Representative hematoxylin and eosin-stained colon sections from the three recipient groups. **c** The number of donor effector T cells in the mLNs was quantified by FACS. **d** The expression of inflammatory cytokines was analyzed in total colonic tissue by qRT-PCR. **e** Percentages of Foxp3^+^ cells among reisolated Thy1.1^+^CD4^+^ WT and *Cd25*^Y129H^ Treg cells 8 weeks after transfer into Rag2^−/−^ recipient mice. **f** Foxp3^+^ and Foxp3^−^ CD4^+^ splenocytes were sorted from WT and *Cd25*^Y129H^ mice, and genomic DNA was analyzed to determine the methylation status of the TSDR. One representative analysis from two independent experiments is shown. **g** Methylation status of the TSDR in reisolated Thy1.1^+^CD4^+^ WT and *Cd25*^Y129H^ Treg cells and naive Thy1.2^+^CD4^+^ T cells from the mesenteric LNs (mLNs) and spleen 8 weeks after transfer into Rag2^−/−^ recipient mice. The percentage of methylation is color-coded according to the scale shown. Data were derived from four (**a**–**d**) and three (**e**, **g**) independent experiments and are represented as the mean ± SD. ns not significant; *p < 0.05, **p < 0.01, ***p < 0.001. One-way ANOVA with Tukey’s correction for multiple comparisons (**a**, **c**) or two-tailed unpaired Student’s *t* test (**d**, **e**)

We next investigated the capacity of *Cd25*^Y129H^ Treg cells to suppress intestinal inflammatory responses in vivo. In an adoptive T-cell transfer colitis model, delivery of naive CD4^+^ T cells into immunodeficient recipients results in severe intestinal inflammation that can be prevented by cotransfer of Treg cells.^47,48^ Adoptive transfer of effector CD25^−^CD62L^+^CD4^+^ WT T cells (naive CD4^+^) into Rag2^−/−^ recipients resulted in the development of colitis with extensive infiltration of inflammatory cells and significant induction of inflammatory cytokines within the colonic tissue (Fig. 8a–d). Notably, cotransfer of naive WT CD4^+^ T cells and *Cd25*^Y129H^ Treg cells into Rag2^−/−^ recipients did not prevent the development of colitis as efficiently as cotransfer of WT Treg cells. Histological analysis of colon samples from recipient mice coinjected with naive WT CD4^+^ T cells and *Cd25*^Y129H^ Treg cells showed significantly increased features of pathological inflammation (Fig. 8a, b) and increased production of inflammatory cytokines compared to recipient mice coinjected with naive WT CD4^+^ T cells and WT Treg cells (Fig. 8d).

Notably, 30% of the *Cd25*^Y129H^ Treg cells in the mLNs exhibited reduced Foxp3 expression 8 weeks after colitis induction, while WT Treg cells largely retained Foxp3 expression (Fig. 8e). This observation suggested that impaired IL-2R signaling by Treg cells significantly affected lineage stability and the suppressive capacity during inflammation. Demethylation of the TSDR is required for stable expression of Foxp3 and thus for stable maintenance of their suppressive capacity. Compared to Treg cells isolated ex vivo, Treg cells induced in vitro show an incomplete demethylation phenotype despite displaying high Foxp3 expression, indicating that the DNA demethylation pattern of the TSDRs serves as a marker of long-term Treg cell stability and function.^49^ To determine whether the instability of adoptively transferred *Cd25*^Y129H^ Treg cells is due to reduced TSDR demethylation, we compared the methylation status of the TSDR in splenic WT and *Cd25*^Y129H^ Treg cells. However, pyrosequencing of bisulfite-treated genomic DNA from sorted Foxp3^+^ and Foxp3^−^ CD4^+^ T cells revealed a similarly demethylated TSDR in Treg cells from WT and *Cd25*^Y129H^ mice (Fig. 8f). Given the role of the TSDR in maintaining Foxp3 expression, these results were not unexpected, since Foxp3 protein levels were barely altered in *Cd25*^Y129H^ Treg cells under steady-state conditions (Fig. 2e). Moreover, this observation confirmed that in Treg cells, a long-term reduction in IL-2R signaling diminishes the size of the Treg cell pool without affecting cell stability. Next, we examined the demethylation of the TSDR in WT and *Cd25*^Y129H^ Treg cells after transfer into Rag2^−/−^ mice. Consistent with the reduced suppressor function of *Cd25*^Y129H^ Treg cells, these cells showed a tendency toward a decrease in TSDR demethylation, although this change was not statistically significant (Fig. 8g). Moreover, this reduction correlated with a decrease in Foxp3 levels, indicative of a role for IL-2R signaling in Treg cell stability.

A seemingly unexpected observation was that *Cd25*^Y129H^ mice did not spontaneously develop autoimmunity, while in the adoptive transfer colitis model, Treg cells from these mice could not prevent autoimmunity. These findings raised the possibility that *Cd25*^Y129H^ Treg cells are able to sufficiently deprive only *Cd25*^Y129H^ effector cells, not WT effector cells, of IL-2, since the latter have higher levels of IL-2R. To test this hypothesis, we performed additional colitis experiments in which Rag2^−/−^ recipients were transferred with naive *Cd25*^Y129H^ CD4^+^ T cells alone or in combination with WT or *Cd25*^Y129H^ Treg cells. In contrast to the above outlined hypothesis, we found that transfer of naive *Cd25*^Y129H^ CD4^+^ T cells resulted in disease with a severity similar to that observed in mice transferred with naïve WT CD4^+^ T cells (Fig. 8 and Supplementary Fig. 8). In both situations, *Cd25*^Y129H^ Treg cells were unable to control inflammation in recipient mice. This finding indicated that the loss of the suppressor function of *Cd25*^Y129H^ Treg cells in the colitis model might be independent of IL-2 deprivation and points to an intrinsic role for IL-2R signaling in suppressing autoimmunity beyond depriving effector cells of IL-2.

## Discussion

Previous reports have demonstrated that IL-2R signaling is required for the development, peripheral maintenance, and suppressor function of Treg cells. However, a severe limitation in studying the role of IL-2R in these processes has been the finding that genetically engineered mice deficient in components involved in IL-2R signaling, including IL-2,^4,13^ IL-2Rα,^4,12,50^ and IL-2Rβ,^11,14^ suffer from early systemic autoimmunity and fundamental alterations in Treg cell development. Therefore, it has been difficult to differentiate between thymic and peripheral defects and between resting and inflammatory conditions. Taking advantage of mice bearing a hypomorphic mutation in the *Cd25* locus, the present study offers novel insights into the roles of CD25 and IL-2R signaling in the homeostasis and stability of Treg cells under steady-state and inflammatory conditions.

In humans, two patients harboring different mutations in the *IL-2Rα* gene that markedly abrogate CD25 cell-surface expression have been identified.^51,52^ One of these mutations leads to an amino acid substitution at codon 166 of the protein (S166),^52^ which is located close to Y129 (Supplementary Fig. 2b), suggesting that mutations in this region may lead to structural rearrangements that affect protein stability. Another reported mutation leads to a substitution of cysteine at codon 51,^51^ which is in close proximity to the site of interaction with IL-2. Notably, in both patients, CD25 was still detectable at low levels in the cytoplasm, thus resembling the phenotype observed in *Cd25*^Y129H^ mice. Interestingly, the lack of surface CD25 led to IPEX-like syndrome, which is characterized by early immune dysfunction and systemic autoimmunity, in both patients, similar to other reported cases with mutations within the IL-2Rα gene that completely impaired CD25 expression.^53,54^ It remains unclear why humans but not *Cd25*^Y129H^ mice develop spontaneous autoimmunity, even though surface levels of CD25 are reduced to a similar degree. It seems possible that the residual expression of CD25 in *Cd25*^Y129H^ mice allows sufficient IL-2R-mediated signaling to prevent autoimmunity. Alternatively, this difference in outcome may reflect higher levels of self-antigen challenge in humans than in inbred mice.

We found a substantial reduction in Treg cell numbers in *Cd25*^Y129H^ mice, indicating that optimal IL-2R signaling is essential for peripheral Treg cell homeostasis, as suggested previously.^3,4,6,50^ However, unlike mice deficient in IL-2 or IL-2Rα,^4,12,13,50^ *Cd25*^Y129H^ mice did not exhibit signs of spontaneous autoimmunity. Our study shows that low IL-2R signaling is sufficient to maintain basal Treg cell numbers with a sufficient suppressive capacity for maintaining immune system homeostasis under noninflamed conditions. One potential limitation of these observations is that the mutation identified has only been investigated in one genetic background. Thus, the interpretation of our results could be extended by studying the *Cd25*^Y129H^ mutation in mice on other genetic backgrounds, including those known to develop spontaneous autoimmunity.

Our findings raise the possibility that mature Treg cells may compensate for low IL-2R signaling by using signals induced by other cytokines, such as IL-7 or IL-15, that may sufficiently preserve the function but not the pool of peripheral Treg cells in a steady state. The lack of autoimmunity in *Cd25*^Y129H^ mice could also be explained by an alternative scenario given that activated T cells and Treg cells are known to regulate each other through IL-2 secretion and consumption, respectively.^10,11,29,55,56^ Therefore, in *Cd25*^Y129H^ mice, impaired IL-2R signaling by autoreactive T cells may lead to decreased IL-2 production, which may be controlled by Treg cells even with low IL-2R activity, thereby maintaining the regulatory loop at a lower functional level. Surprisingly, the lack of spontaneous autoimmunity was not reflected in the in vitro Treg cell suppression assay, which revealed a strong dependency on IL-2R signaling for Treg cell suppressive function in vitro, as previously suggested.^9,10^ This observation indicates that commonly used in vitro coculture suppression assays more likely mimic the requirements for Treg cell function in inflammatory states than in steady-state situations.

Under steady-state conditions, *Cd25*^Y129H^ Treg cells maintained Foxp3 expression, although at a moderately lower level, consistent with previous results where sustained Foxp3 expression was observed upon IL-2 deprivation with anti-IL-2 neutralizing antibodies.^6,16^ Reduced Foxp3 expression was accompanied by the acquisition of an effector phenotype but did not compromise Treg cell identity or lead to the formation of “exFoxp3^+^ cells.”^57^

In contrast, our results from the adoptive transfer colitis model demonstrate that efficient IL-2R signaling is important for Treg cell stability and function during inflammation, implying that IL-2Rα-independent signals cannot compensate for low IL-2R signaling under such settings. Notably, using *Cd25*^Y129H^ mice allowed us to specifically evaluate the role of IL-2R signaling in Treg cell stability under inflammatory conditions without the intrinsic lymphoproliferative disorder associated with germline depletion of genes involved in the IL-2R signaling machinery, which might explain the diverse results reported in the past.^57–59^ Here, we showed that reduced CD25 expression caused severe defects in Treg cell stability. Approximately 30% of Treg cells lost Foxp3 expression after colitis induction, indicating that efficient IL-2R signaling is required for sustained Foxp3 expression and suppressive function under inflammatory conditions. Loss of Foxp3 expression and subsequent Treg cell stability could be due to exposure to proinflammatory cytokines^58,60^ or deprivation of IL-2 signals. It has been previously shown that signaling via IL-2R sustains Foxp3 expression by counteracting proinflammatory cytokine-driven Foxp3 silencing mechanisms at the level of the TSDR.^61^ The present study supports and extends this view. Here, methylation analysis of the TSDR did not reveal any differences between WT and *Cd25*^Y129H^ splenic Treg cells, indicating that the hypomorphic *Cd25*^Y129H^ mutation provides sufficient signals for maintaining TSDR demethylation under physiological conditions. However, once Treg cells reside in inflamed tissue, they rely on fully functional IL-2R signaling machinery to maintain Foxp3 expression and the functional phenotype.

The assumption that IL-2R signaling is essential for Treg cell development in the thymus is based on the observation that mice genetically deficient in IL-2, IL-2Rα, or IL-2Rβ exhibit systemic lethal autoimmunity, which has been ascribed primarily to reduced frequencies of thymic Treg cells.^3,25,50,62^ Alternatively, other studies suggest that thymic Treg cell development may additionally require other cytokines because there is still a substantial proportion of thymic Treg cells in IL-2- or IL-2Rα-deficient mice.^4,19^ In addition to IL-2, other cytokines, such as primarily IL-7 and IL-15 but also IL-4, IL-9, and IL-21, that signal through the common γ-chain may also contribute to thymic Treg cell development because deficiency in the common γ-chain results in complete absence of thymic Treg cells even though their availability within the thymus may be limiting.^4,63–65^ In the present study, decreased CD25 expression in *Cd25*^Y129H^ mice did not lead to significant reductions in the percentage or absolute number of thymic Treg cells, indicating that unimpaired IL-2R signaling is less important for thymic Treg cell development than for peripheral Treg cell homeostasis. scRNA-seq recently revealed a considerable degree of heterogeneity in the Treg cell compartment in various lymphoid organs and NLTs.^33,45,66^ In line with these studies, the scRNA-seq data in the present study identified four major Treg cell subsets showing characteristic cell activation phenotypes and gene transcription signatures, as well as different expression patterns for genes regulating migration and adhesion.

Previously, Stat-1^+^ Treg cells were exclusively identified in the brachial LNs, but not in the spleen or other organs investigated.^33^ In contrast, the present study identified this Treg cell population in the spleen using nonsupervised hierarchical cluster analysis, which might be due to technical details, such as differences in sequencing or sample processing. Nevertheless, the fact that this Treg subpopulation can be found in different lymphoid tissues suggests that their development is governed by external stimuli that are not restricted to a specific environment. Moreover, the fact that Stat-1^+^ Treg cells showed increased expression of IFN-related molecules suggests that they represent an eTreg cell subset that may arise from the de novo conversion of conventional CD4^+^CD25^−^ T cells into Treg cells;^67–69^ however, this hypothesis still needs to be clarified.

Notably, compared to WT Treg cells, *Cd25*^Y129H^ Treg cells were grouped rather differently, highlighting the impact of IL-2R signaling on the organization of the Treg cell compartment. Our data show that efficient IL-2R signaling is particularly required for the maintenance of cTreg cells since we observed a decrease in the absolute number of CD62L^+^CD44^−^ Treg cells but not in that of CD62L^−^CD44^+^ Treg cells. Our findings also suggest that long-lasting impairment in IL-2R signaling may reduce the stability of cTreg cells, which could subsequently transform into eTreg cells.

A previous study reported that IL-2R signaling controls chromatin accessibility and the epigenetic landscape of thymic precursor Treg cells in the same mouse model that was used in the present study.^70^ The results of that study were in agreement with those of earlier reports suggesting that IL-2R regulates gene expression by modulating chromatin conformation at specific transcriptional enhancers.^71,72^ Along with those findings, our observations indicate that optimal IL-2R signaling is required for Treg cell identity and stability and thus for optimal Treg cell function. Further studies will be needed to determine the precise mechanisms by which IL-2R signaling promotes Treg cell stability in different environments and whether these mechanisms can be targeted to modulate Treg cell function to dampen inflammatory diseases.

Finally, the scientific community should be reminded that the *Cd25*^Y129H^ mutation spontaneously occurred in a *Ccr2*^−/−^ mouse strain that has been widely used to study the role of the chemokine receptor CCR2 in the immune system. Since when this mutation occurred is unknown, it might be appropriate to revisit at least those experiments that studied T-cell responses using *Ccr2*^*tm1Ifc*^ mice.

### Supplementary information

Supplementary Table 1

Supplementary Table 2

Supplementary Table 3

Supplementary Table 4

Supplementary Table 5

### Supplementary information

Supplementary Figures

## Data Availability

The scRNA-seq data that support the findings of this study have been submitted to the National Center for Biotechnology Information/Gene Expression Omnibus database (https://www.ncbi.nlm.nih.gov/geo) under accession number GSE161345. The source data underlying Figs. 1c, e–h, 2b–e, 3a–h, 4a, b, d, 6a, b, 7b, and 8a, c–e, g, and Supplementary Figs. 3a–c, 4a–e, 5a, b, 6, and 8a, b are provided as a Source Data file. All other data that support the findings of this study are available from the corresponding author upon reasonable request.

## Source data

Source Data
