# Supplementary material for: Efficient IL-2R signaling differentially affects the stability, function, and composition of the regulatory T-cell pool
Source: Cell Mol Immunol. 2021 Jan 6;18(2):398–414. doi: 10.1038/s41423-020-00599-z (PMC8027001; doi:10.1038/s41423-020-00599-z)
Supplement: Supplementary file 6 — Supplementary Figures [file 41423_2020_599_MOESM7_ESM.docx]

**Efficient IL-2R signaling differentially affects stability, function, and composition of the regulatory T cell pool**

Marc Permanyer^1^, Berislav Bošnjak^1^, Silke Glage^2^, Michaela Friedrichsen^1^, Stefan Floess^3^, Jochen Huehn^3,4^, Gwendolyn E. Patzer^1^, Ivan Odak^1^, Nadine Eckert^1^, Razieh Zargari^1^, Laura Ospina-Quintero^1^, Hristo Georgiev^1^ & Reinhold Förster^1,4^

^1^ Institute of Immunology, Hannover Medical School, Hannover, Germany

^2^ Institute for Laboratory Animal Science, Hannover Medical School, Hannover, Germany

^3^Department Experimental Immunology, Helmholtz Centre for Infection Research, Braunschweig, Germany

^4^ Cluster of Excellence RESIST (EXC 2155), Hannover Medical School, 30625 Hannover, Germany

**Supplementary information**

Supplementary figures 1 – 8


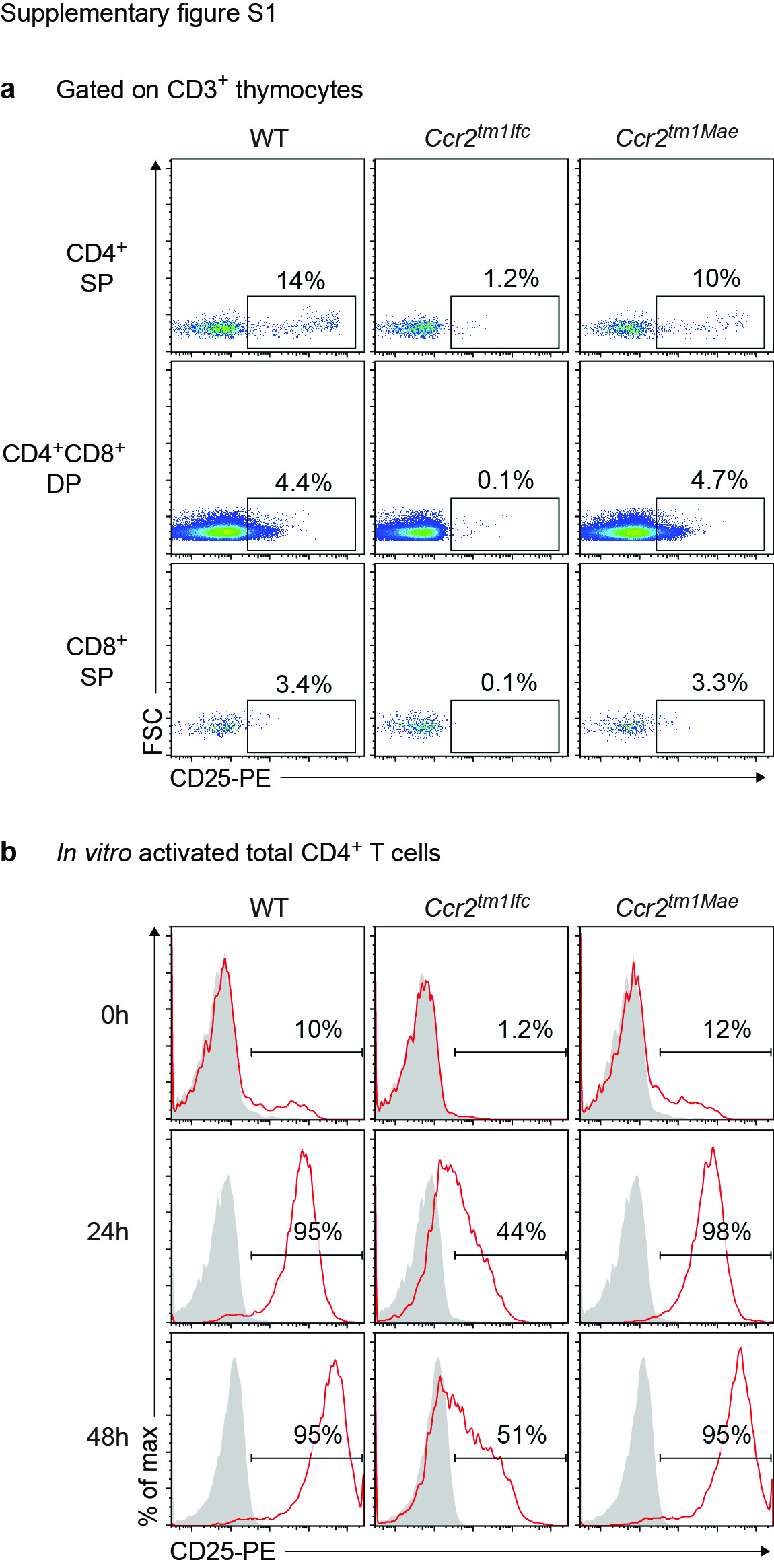


**Supplementary figure 1. Expression of CD25 differs in different *Ccr2* knockout mouse strains.** (**a**) Representative histograms showing percentages of CD25^+^ cells among CD4^+^ and CD8^+^ single-positive (SP) and CD4^+^CD8^+^ double-positive (DP) thymocytes from WT, *Ccr2^tm1Ifc^* and *Ccr2^tm1Mae^* mice as indicated. (**b**) Representative histograms showing percentages of CD25^+^ cells among total CD4^+^ T cells in non-activated (0h) and following anti-CD3/CD28 *in vitro* activation (24h and 48h) from WT, *Ccr2^tm1Ifc^* and *Ccr2^tm1Mae^* mice as indicated. Data are the representative of two independent experiments with 2 mice per genotype.


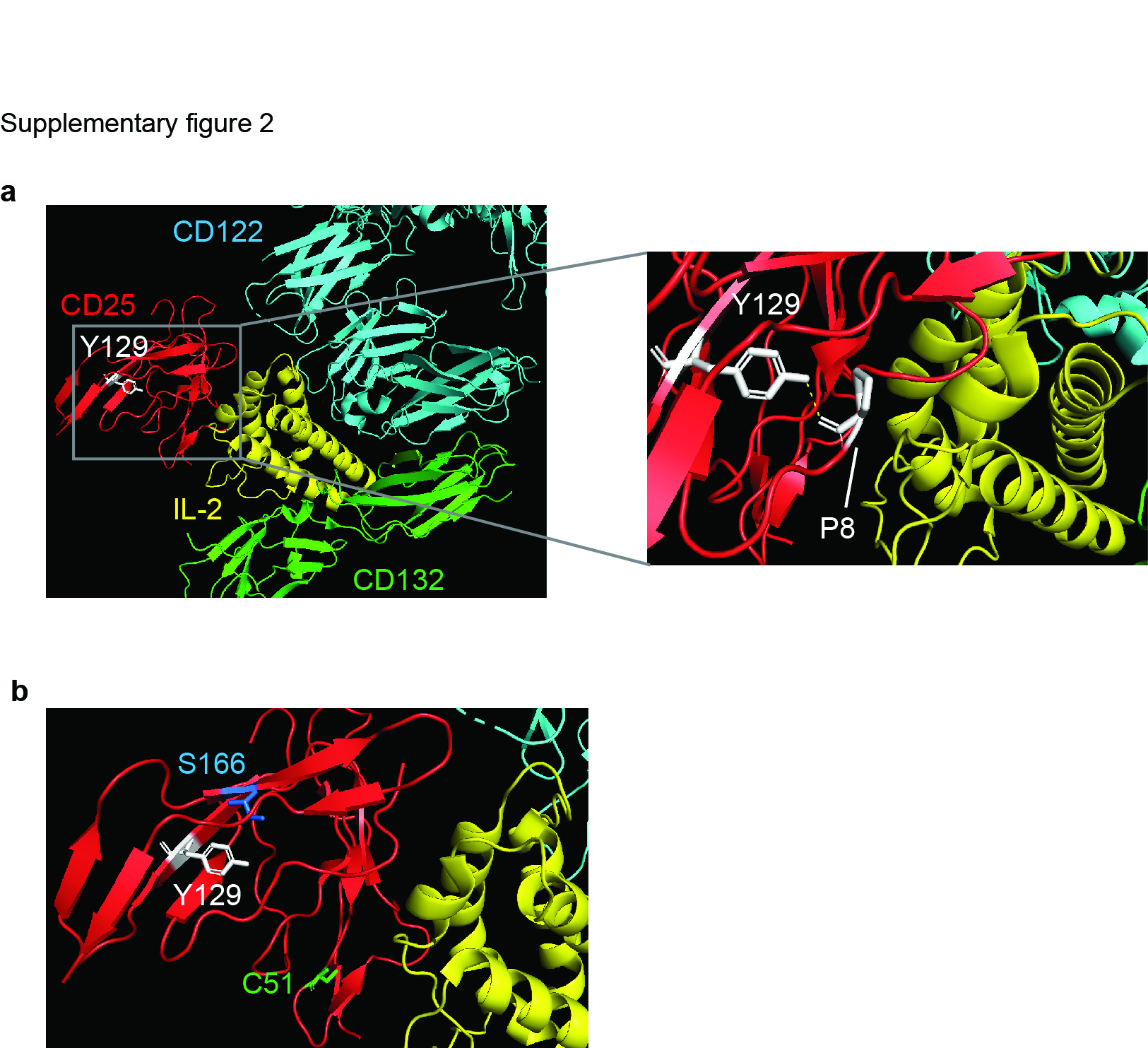


**Supplementary figure 2. Depiction of residues affected by different point mutations of CD25.** (**a**) Localization of the amino acid Y129 (white) based on the solved 3D structure of the human heterotrimeric IL-2R in complex with IL-2 (PDB: 2ERJ). The putative hydrogen bonds formed between Y129 and P8 are shown by a dashed yellow line. (**b**) 3D structure and localization of Y129 (white), S166 (blue) and C51 (green) that are affected in known mutations of human CD25.


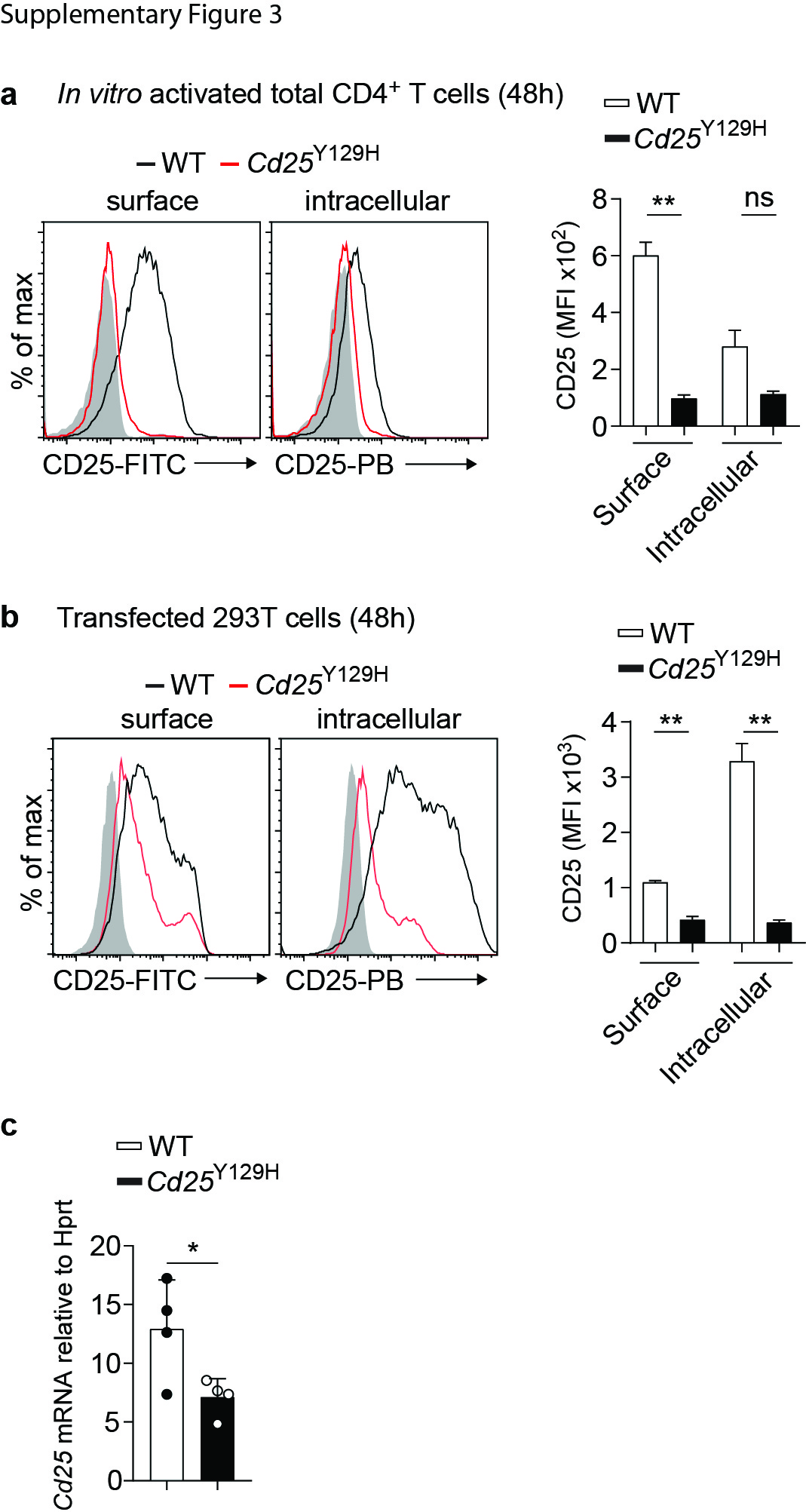


**Supplementary figure 3. *Cd25*^Y129H^ cells show reduced levels of surface and intracellular expression of CD25*.*** (**a**) Representative histograms (left) and quantitative analysis (right) showing expression of surface and intracellular CD25 in total CD4^+^ T cells following *in vitro* activation with anti-CD3/CD28 antibodies for 48 hrs from WT and *Cd25*^Y129H^ mice. (**b**) Representative histograms (left) and quantitative analysis (right) showing expression of surface and intracellular CD25 of 293T cells transiently transfected with a plasmid encoding wildtype Cd25 (black line) or the mutant *Cd25*Y129H (red line). (**c**) RT-PCR analysis of *Cd25* mRNA expression levels in CD4^+^ T cells following *in vitro* activation for 48h with anti-CD3/CD28 antibodies from WT (white bar) and *Cd25*^Y129H^ (black bar) mice. Data are the mean ± SD from two independent experiments with 2 (**a,b**) or 4 (**c**) mice per genotype analyzed. ns, not significant; *p < 0.05, **p < 0.01 (two-tailed unpaired Student *t* test).


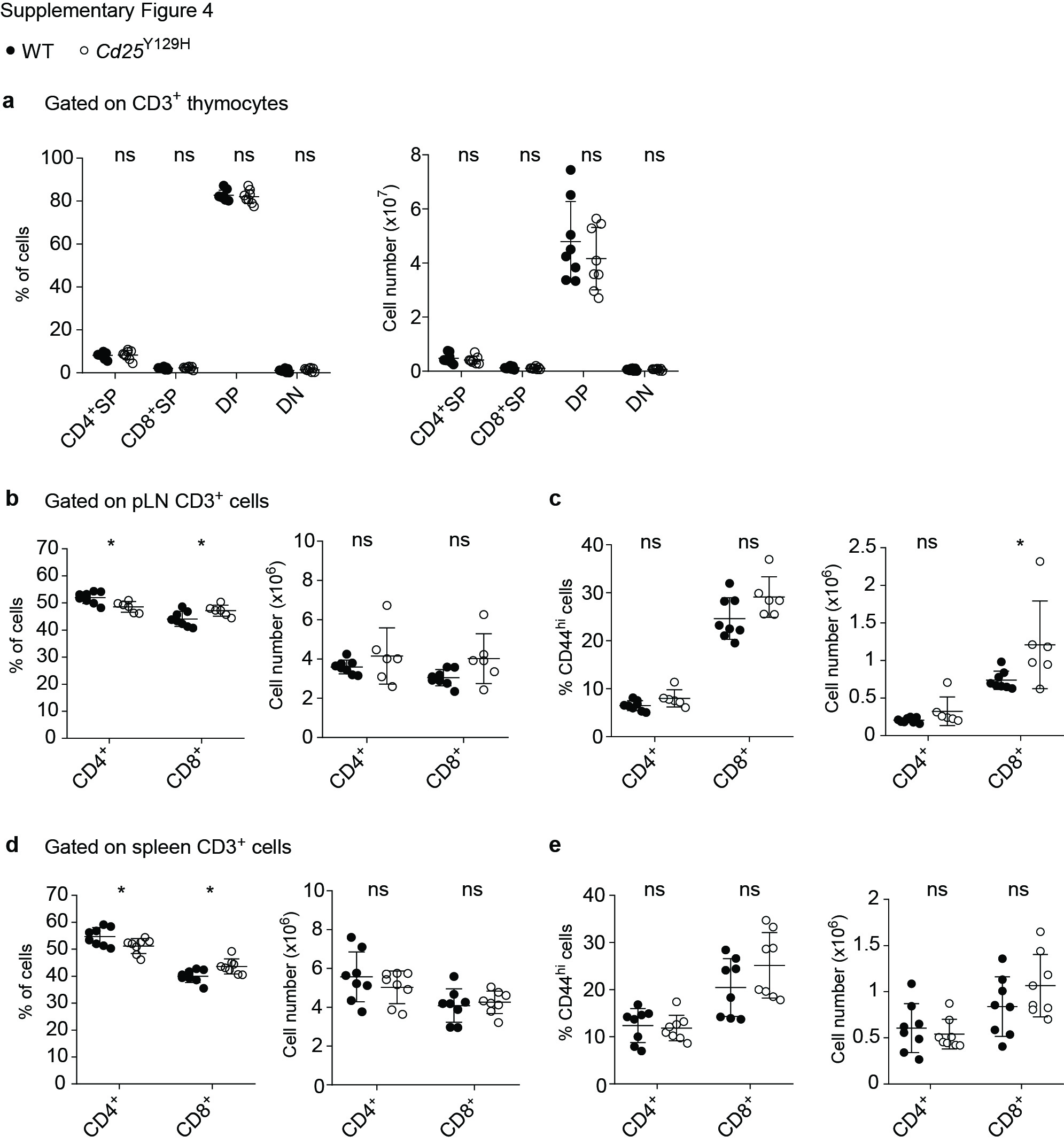


**Supplementary figure 4. Characterization of thymocyte and lymphocyte subpopulations from WT and *Cd25*^Y129H^ mice.** (**a**) Percentages (left) and absolute cell numbers (right) of CD4^+^ and CD8^+^ single-positive (SP), CD4^+^CD8^+^ double-positive (DP) and double-negative (DN) among CD3^+^ thymocytes from WT (filled circles) and *Cd25*^Y129H^ (open circles) mice. (**b**) Percentages (left) and absolute cell numbers (right) of CD4^+^ and CD8^+^ T cells among pLN CD3^+^ cells. (**c**) Percentages (left) and absolute cell numbers (right) of CD44^hi^ cells among pLN CD4^+^ and CD8^+^ T cells. (**d**) Percentages (left) and absolute cell numbers (right) of CD4^+^ and CD8^+^ T cells among spleen CD3^+^ cells. (**e**) Percentages (left) and absolute cell numbers (right) of CD44^hi^ cells among spleen CD4^+^ and CD8^+^ T cells. Data are derived from at least three experiments using 8- to 12-week old mice with at least 6 to 8 mice per genotype. Data are the mean ± SD. ns, not significant; *p < 0.05 (two-tailed unpaired Student *t* test).


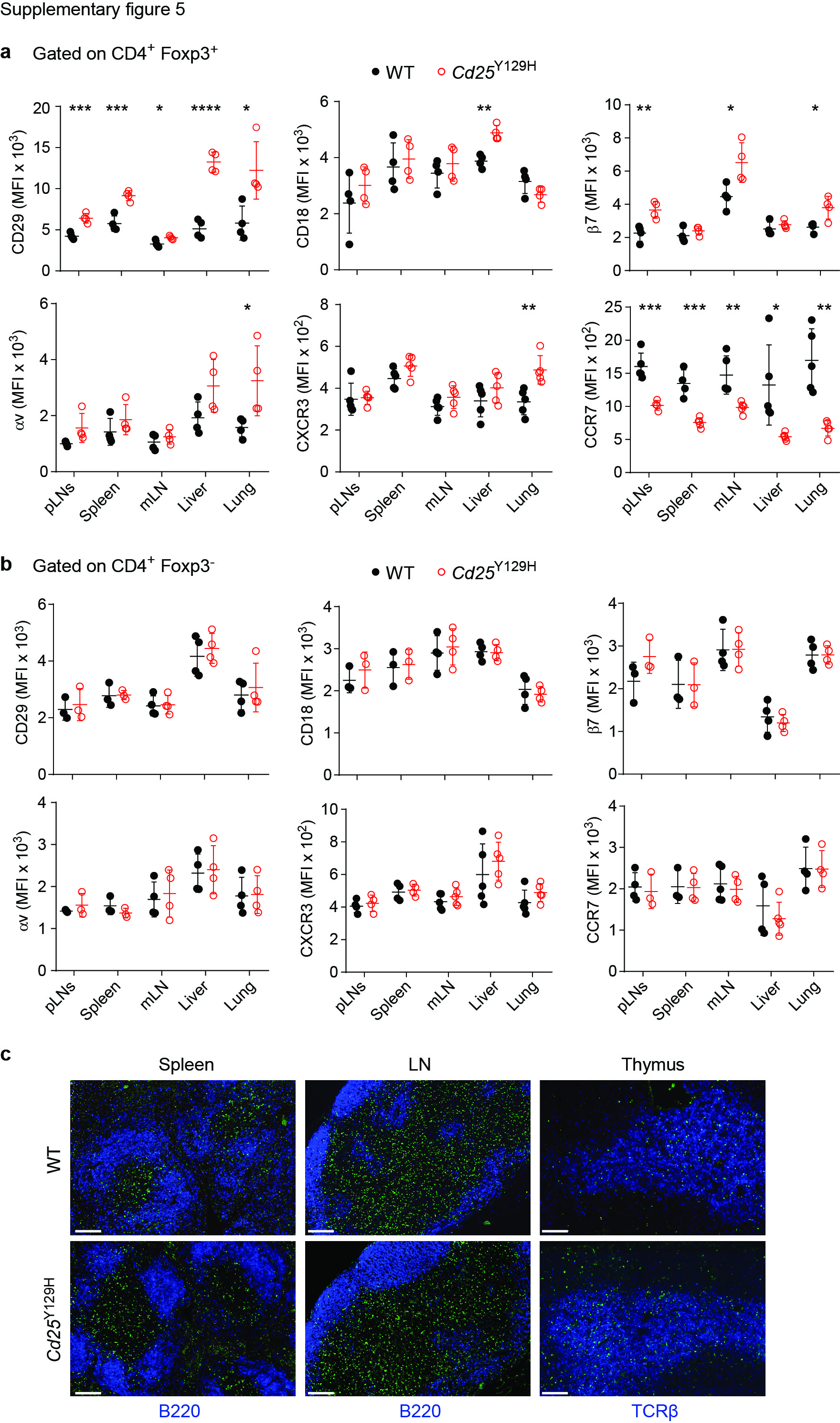


**Supplementary figure 5.** **Effects of IL-2R signaling on the phenotype and localization of Treg cells.** The expression levels of indicated molecules in CD4^+^Foxp3^+^ (**a**) and CD4^+^Foxp3^-^ (**b**) cells of WT (black) and *Cd25*^Y129H^ (red) mice. Data are derived from four independent experiments with 4 – 5 mice per genotype and are represented as mean ± SD; *p < 0.05, **p < 0.01, ***p < 0.001, ****p< 0.0001; two-tailed unpaired Student t test. (**c**) Histology sections of spleen (left), lymph node (LN; middle), and thymus (right) of WT and *Cd25*^Y129H^ mice stained with B220 or TCRβ antibodies to depict the B cell follicles (spleen and LN) and thymic medulla, respectively. One representative image of two independent experiments is shown. Scare bar 100μm.


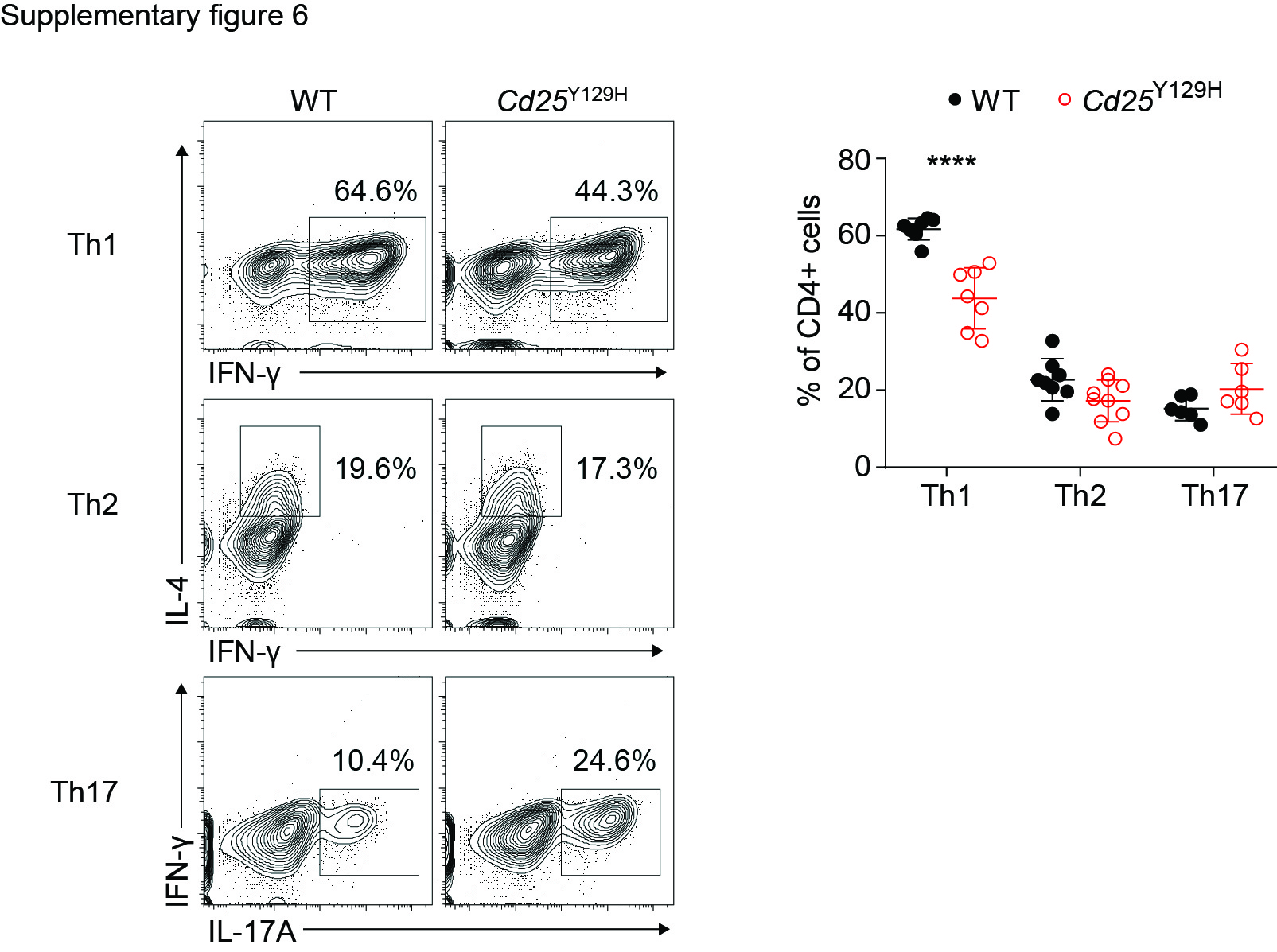


**Supplementary figure 6. Reduced Th1 cytokine responses in *Cd25*^Y129H^ mice.** Representative FACS plots (**a**) and quantitative analysis (**b**) of indicated intracellular cytokines upon stimulation of WT and *Cd25*^Y129H^ conventional CD4^+^ T cells under Th1, Th2, and Th17-polarizing conditions for 5 days. Data are derived from four independent experiments with 6 – 8 mice per genotype and are represented as mean ± SD; ****p< 0.0001; two-tailed unpaired Student t test.


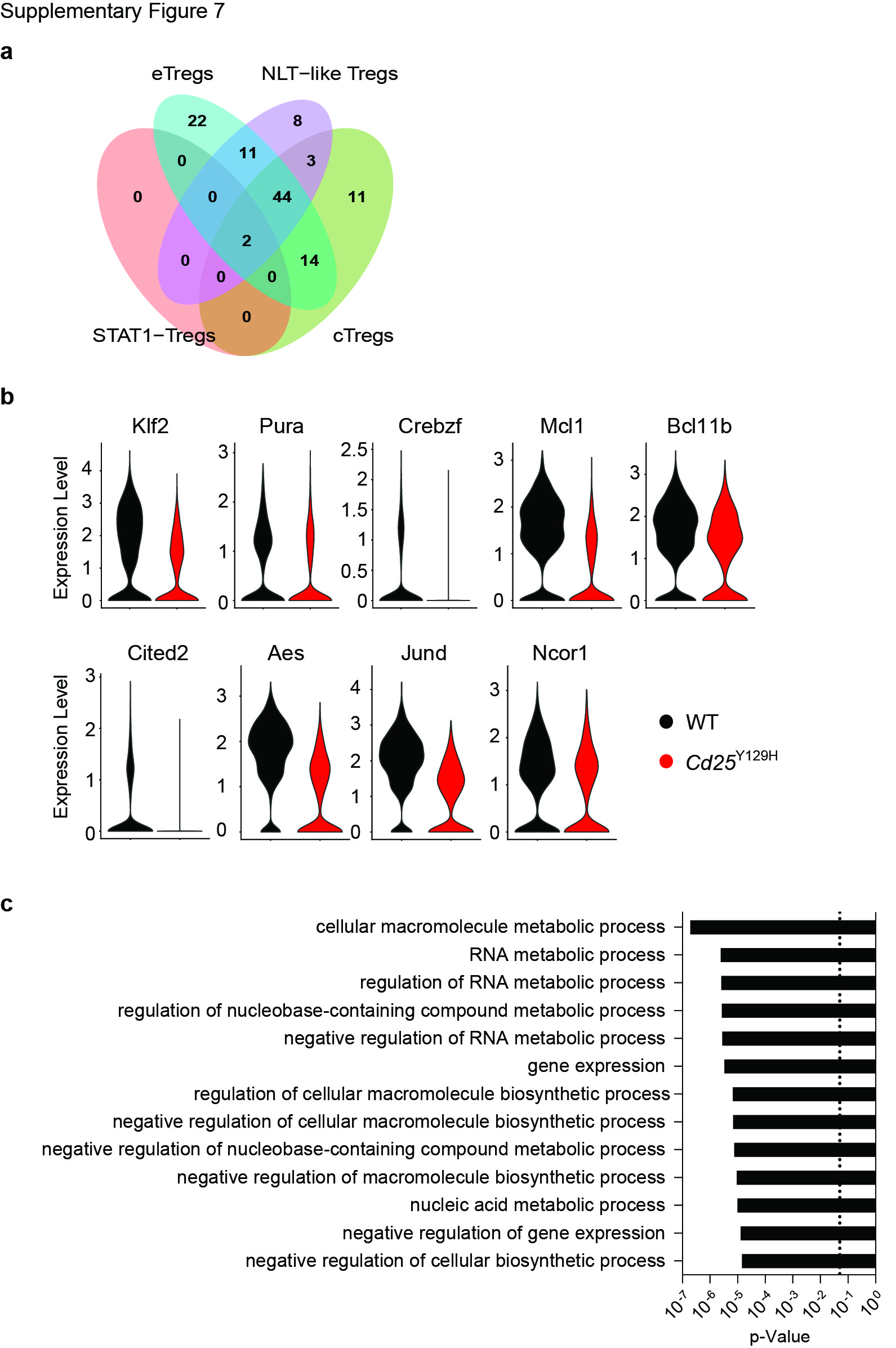


**Supplementary figure 7. Differential regulation of gene expression and biosynthesis in *Cd25*^Y129H^ Treg cells.** (**a**) Venn diagram showing the overlap between clusters 1, 2, 3, and 4, from genes that were upregulated or downregulated in WT Treg cells compared with *Cd25*^Y129H^ Treg cells. (**b**) Violin plots displaying expression of selected genes in WT or *Cd25*^Y129H^ Treg cells. (**c**) Top 10 gene ontology (GO) Biological Process terms significantly enriched in WT Treg cells. GO terms are listed in order of statistical significance. Cut-off for selection was FDR < 0.01 and fold enrichment > 2.5.


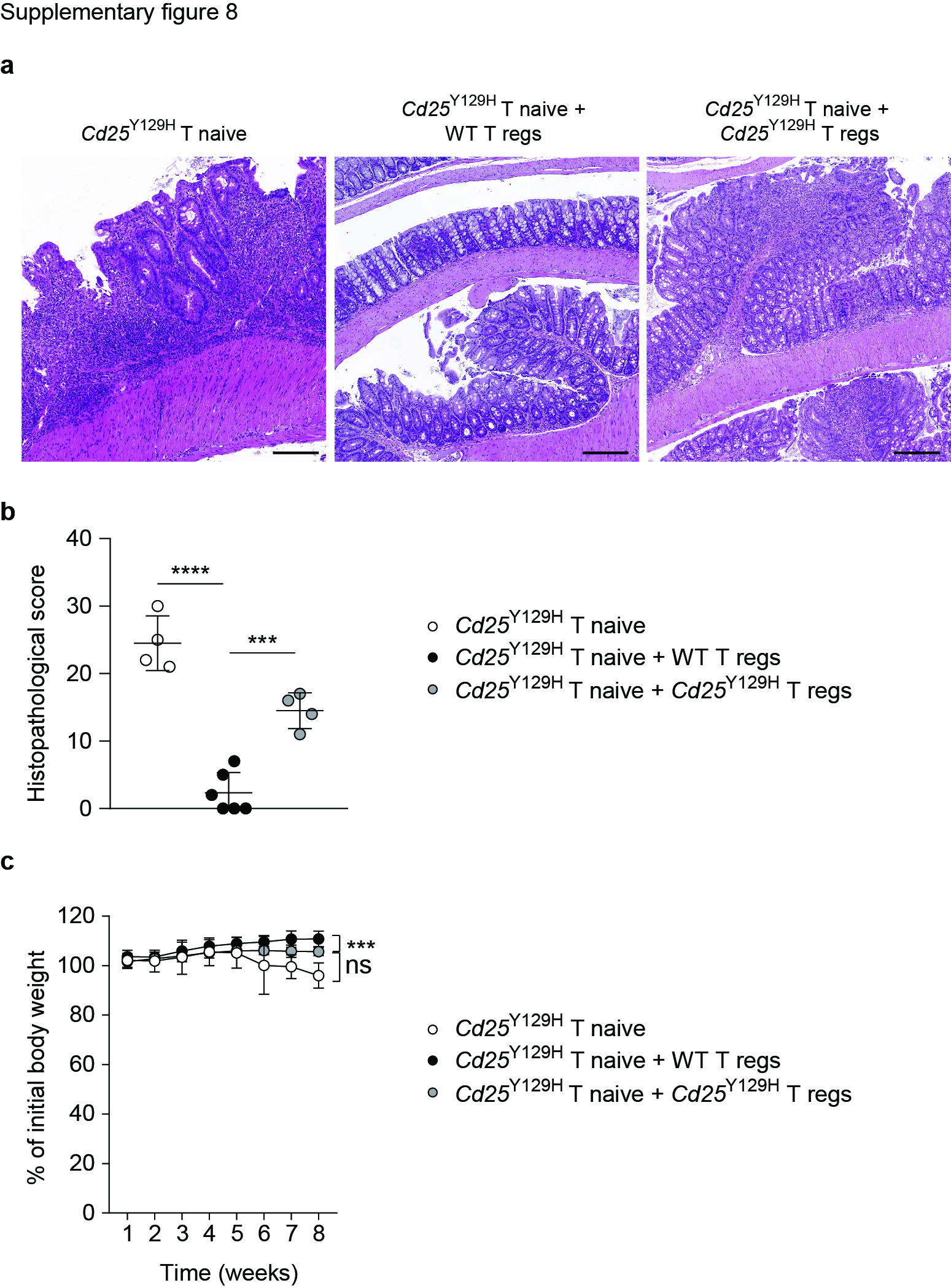


**Supplementary figure 8. Effector *Cd25*^Y129H^ T cells induce similar colitis disease as observed with effector WT T cells.** (**a**) Representative hematoxylin and eosin staining and (**b**) histopathological scoring of colons from Rag2^-/-^ mice injected with *Cd25*^Y129H^ naïve CD25^-^CD62L^+^CD4^+^ T cells alone (open circles), or together in combination with WT (filled circles) or *Cd25*^Y129H^ (grey circles) GFP^+^Foxp3^+^ Treg cells. (**c**) Body weight of mice transferred with the indicated cell combinations. Data are derived from two independent experiments with 4 – 6 mice per genotype and are represented as mean ± SD. ns, not significant; ***p < 0.001, ****p< 0.0001 One-way (**a**) and Two-way (**b**) ANOVA with Tukey´s correction for multiple comparisons.
